# Comparative fecal metagenomics unveils unique functional capacity of the swine gut

**DOI:** 10.1186/1471-2180-11-103

**Published:** 2011-05-15

**Authors:** Regina Lamendella, Jorge W Santo Domingo, Shreya Ghosh, John Martinson, Daniel B Oerther

**Affiliations:** 1 University of Cincinnati, Department of Civil and Environmental Engineering, Cincinnati, OH 45220, USA; 2 National Risk Management Research Laboratory, U. S. Environmental Protection Agency, Cincinnati, OH 45268, USA; 3 National Exposure Research Laboratory, U. S. Environmental Protection Agency, Cincinnati, OH 45268, USA; 4 Lawrence Berkeley National Laboratory, Earth Sciences Division, Berkeley, CA 94720, USA; 5 Department of Civil and Environmental Engineering, Missouri University of Science and Technology, Rolla, MO 65409, USA

## Abstract

**Background:**

Uncovering the taxonomic composition and functional capacity within the swine gut microbial consortia is of great importance to animal physiology and health as well as to food and water safety due to the presence of human pathogens in pig feces. Nonetheless, limited information on the functional diversity of the swine gut microbiome is available.

**Results:**

Analysis of 637, 722 pyrosequencing reads (130 megabases) generated from Yorkshire pig fecal DNA extracts was performed to help better understand the microbial diversity and largely unknown functional capacity of the swine gut microbiome. Swine fecal metagenomic sequences were annotated using both MG-RAST and JGI IMG/M-ER pipelines. Taxonomic analysis of metagenomic reads indicated that swine fecal microbiomes were dominated by Firmicutes and Bacteroidetes phyla. At a finer phylogenetic resolution, *Prevotella *spp. dominated the swine fecal metagenome, while some genes associated with *Treponema *and *Anareovibrio *species were found to be exclusively within the pig fecal metagenomic sequences analyzed. Functional analysis revealed that carbohydrate metabolism was the most abundant SEED subsystem, representing 13% of the swine metagenome. Genes associated with stress, virulence, cell wall and cell capsule were also abundant. Virulence factors associated with antibiotic resistance genes with highest sequence homology to genes in Bacteroidetes, Clostridia, and *Methanosarcina *were numerous within the gene families unique to the swine fecal metagenomes. Other abundant proteins unique to the distal swine gut shared high sequence homology to putative carbohydrate membrane transporters.

**Conclusions:**

The results from this metagenomic survey demonstrated the presence of genes associated with resistance to antibiotics and carbohydrate metabolism suggesting that the swine gut microbiome may be shaped by husbandry practices.

## Background

The animal gastrointestinal tract harbors a complex microbial network and its composition reflects the constant co-evolution of these microorganisms with their host environment [1]. Uncovering the taxonomic composition and functional capacity within the animal gut microbial consortia is of great importance to understanding the roles they play in the host physiology and health. Since animal feces can harbor human pathogens, understanding the genetic composition of fecal microbial communities also has important implications for food and water safety. The structure and function of the gut microbial community has received significant attention for decades, although most of the work was restricted by the use of culture-based techniques. Recently, sequence analysis of the 16S rRNA gene has shed new light on the diversity and composition of microbial communities within several animal gut systems [2]. While 16S rRNA gene-based techniques have revealed impressive microbial diversity within gut environments, this approach offers only limited information on the physiological role of microbial consortia within a given gut environment.

Random sequencing of metagenomes has allowed scientists to reveal significant differences in metabolic potential within different environments [3], including microbial populations associated with host-microbial partnerships. Specifically, the publicly available database IMG/M [4] contains 596 Mb of sequencing data, representing 1,424, 000 genes from 17 different gut microbiomes. Studying gut metagenomes has particularly helped in uncovering several important biological characteristics of these microbiomes. For example, when 13 human gut metagenomes were compared, Kurokawa et al [5] found that adult and infant type gut microbiomes have enriched gene families sharing little overlap, suggesting different core functions within the adult and infantile gut microbiota. This study also demonstrated the presence of hundreds of gene families exclusively found in the adult human gut, suggesting various strategies are employed by each type of microbiota to adapt to its intestinal environment [5]. Other gut microbiome studies support these significant differences in core and variable gene content from different animal hosts and environments [1,6-12]. Thus, comparing the gene content of multiple gut microbiomes can help elucidate the ecological underpinnings of gut systems.

Thus far, the functional genetic potential of the pig distal gut microbiota has not been studied using metagenomics, although it is reasonable to assume that the swine gut supports similar genetic complexity to the human gut system, as they both prefer omnivorous feeding behavior and harbor similar bacterial groups as determined by several phylogenetic studies [13-15]. In this study we used metagenomic data analyses to characterize the swine fecal microbiome with respect to species composition and functional content. In order to search for the potential presence of unique gene functions harbored by the swine gut microbiome, we performed a comparative metagenomic approach, in the context of phylogenetic and functional composition.

## Results

### Taxonomic distribution of swine fecal metagenomic sequences

**Figure 1:**
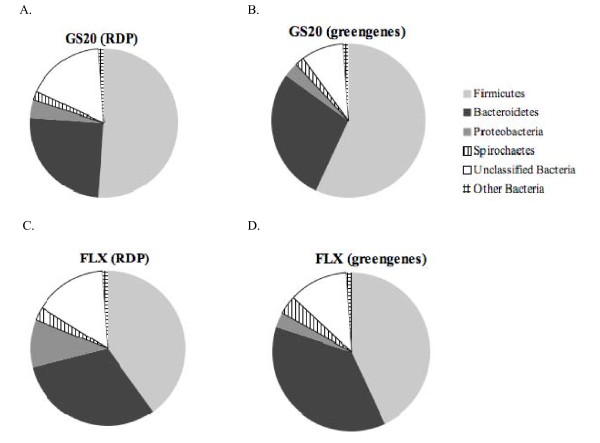
**Taxonomic composition of bacterial phyla using 16S rRNA gene sequences retrieved from GS20 and FLX swine fecal metagenomes**. Using the "Phylogenetic Analysis" tool within MG-RAST, the GS20 and FLX sequencing runs were searched against the RDP and greengenes databases using the BLASTn algorithm. The percent of sequences assigned to each of the bacterial phyla from the pig fecal GS20 (A and B) and FLX (C and D) metagenomes is shown. The e-value cutoff for 16S rRNA gene hits to RDP and greengenes databases was 1×10^-5 ^with a minimum alignment length of 50 bp.

Approximately 130 Mb of swine fecal metagenome sequence data were retrieved using two different pyrosequencing platforms (454 GS20 and FLX), making this study the first metagenomic survey of the swine gut (Table 1). The average read length for the GS20 and FLX runs were 156 bp and 230 bp, respectively. Taxonomic distribution of 16S rRNA gene sequences from the GS20 and FLX swine fecal metagenomes revealed similar taxonomic distributions (Figure 1). However, some differences in classification of 16S rRNA genes retrieved from the GS20 and FLX runs were noted. Most interestingly, fewer Firmicutes and more Bacteroidetes were classified using the FLX 16S rRNA genes (using both RDP and Greengenes databases). This finding suggests shorter read lengths may lead to misclassification of these two divergent phyla. Additionally, more unclassified sequences were retrieved from GS20 metagenomic reads with e-values less than 0.01.

**Table 1:** Summary of pyrosequencing data from Yorkshire swine fecal samples

	Yorkshire Pig Fecal Metagenome GS20	Yorkshire Pig Fecal Metagenome FLX
**Total no. of sequences**	157,221	462,501
**Total sequence size (bp)**	24,518,676	106,193,719
**Average sequence length (bp)**	155.95	229.61
Genes*	42677	124684
CDS*	42349 (99.23%)	124050 (99.49%)
RNA*	328 (.77%)	634 (.51%)
rRNA*	328	634
5S	25	46
16S	114	248
18S	1	2
23S	181	325
28S	1	3
**Ribosomal Database Project 16S rDNA hits**	328 (0.21%)	1100 (.24%)
**Greengenes 16S rDNA hits**	295 (0.19%)	912 (0.20%)
w/Func Prediction*	33249 (77.9%)	93804 (75.2%)
COG*	33997 (79.7%)	97053 (77.8%)
Pfam*	34589 (81.0%)	99027 (79.4%)
TIGRfam*	16117 (37.8%)	44040 (35.3%)
Genome Properties*	3881 (9.1%)	10599 (8.5%)
Signalp*	11125 (26.1%)	35780 (28.7%)
TransMb*	8863 (20.8%)	26949 (21.6%)
MetaCyc*	3694 (8.7%)	10815 (8.7%)

* Indicates that these summary statistics were generated using the IMG/M-ER annotation system offered through the Joint Genome Institute [4] using the proxygene method [34].

Both GS20 and FLX metagenomic swine fecal datasets were dominated by Firmicutes and Bacteroidetes phyla (Figure 1), which is consistent with several molecular phylogenetic studies of mammalian gut environments, including the swine gut [2,8,10,14]. Archaeal sequences constituted less than 1% of total rRNA gene sequences retrieved in either swine metagenome, and were dominated by the *Methanomicrobia *and *Thermococci*, which is consistent with previous molecular diversity studies of pig manure [16]. While these populations are only a very small fraction of the total microbiota [17], methanogens contribute significantly to the metabolic potential within in a gut environment [18]. The majority of eukaryotic sequences derived from the swine metagenomes are related to Chordata (i.e., host phylum), fungi, and the Viridiplantae (i.e., feed). Sequences sharing high sequence homology to *Balantidium coli *were obtained in both swine metagenomes. The latter is a protozoan pathogen that causes balantadiasis in mammalian hosts, including human and swine. Since the samples were collected from healthy animals, these sequences might be associated with non-pathogenic *B. coli *strains or with pathogenic strains in asymptomatic animals. Viral sequences were rare, comprising less than 1% of the total metagenomic sequences when compared to the SEED database (Additional File 1, Fig. S1). The low abundance of viral sequences retrieved from the swine fecal metagenomes is consistent with viral proportions retrieved in termite, chicken, and cattle gastrointestinal metagenomes, and may be a direct result of limited representation of viral genetic information in currently available databases [8].

A closer look at the taxonomic distribution of the numerically abundant bacterial orders derived from the swine metagenomes revealed that *Clostridiales*, unclassified Firmicutes, *Bacteroidales*, *Spirochaetales*, unclassified gammaproteobacteria, and *Lactobacillales *were the top six most abundant bacterial groups (Additional File 1, Fig. S2). At the genus-level taxonomic resolution, *Prevotella *species were the most abundant, comprising 19-22% of 16S rRNA gene sequences within both swine fecal metagenomes (Additional File 1, Fig. S3). Of the classified *Clostridiales*, *Sporobacter *was the next most abundant genus within both the swine fecal metagenomic datasets. *Anaerovibro*, *Clostridium*, and *Streptococcus *genera encompassed at least 5% of rRNA gene sequences in either the GS20 or FLX datasets.

### Comparative gut metagenomics using 16S rRNA gene sequences

**Figure 2:**
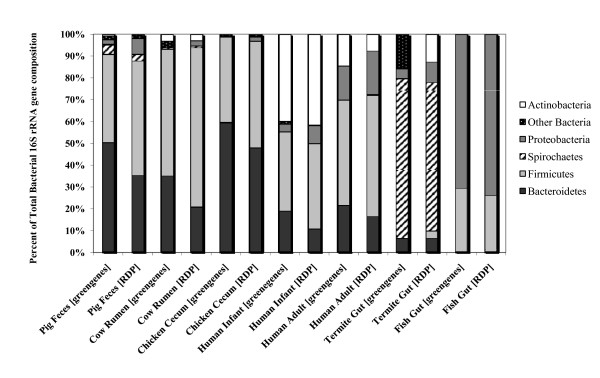
**Taxonomic distribution of bacterial phyla from swine and other currently available gut microbiomes within MG-RAST**. The percent of sequences assigned to each bacterial order from swine and other gut metagenomes is shown. Using the "Phylogenetic Analysis" tool within MG-RAST, each gut metagenome was searched against the RDP and greengenes databases using the BLASTn algorithm. The percentage of each bacterial phlya from swine, human infant, and human adult metagenomes were each averaged since there was more than one metagenome for each of these hosts within the MG-RAST database. The e-value cutoff for 16S rRNA gene hits to the RDP and greengenes databases was 1×10^-5 ^with a minimum alignment length of 50 bp.

We performed comparative metagenomics on 16S rRNA gene sequences derived from publicly available gut metagenomic datasets to reveal phylotype differences between mammalian, avian, and invertebrate distal gut microbiomes. The distribution of bacterial phyla from swine feces appeared closest to that of the cow rumen and chicken cecum, sharing more similar proportions of Bacteroidetes, Firmicutes, Proteobacteria, and Actinobacteria (Figure 2). A statistical analysis comparing bacterial distribution between hosts revealed several significantly different bacterial groups. (Additional File 2, Table S1 and S2). Human adult and infant distal gut microbiomes had significantly higher abundances of Actinobacteria (p < 0.05) than did the swine microbiome (Additional File 2, Table S2). The fish gut microbiome was comprised mostly of Proteobacteria and Firmicutes, while the termite gut was dominated by Spirochetes. Interestingly, the swine fecal metagenome also harbored significantly more Spirochetes than many other hosts. (Additional File 2, Table S3).

**Figure 3:**
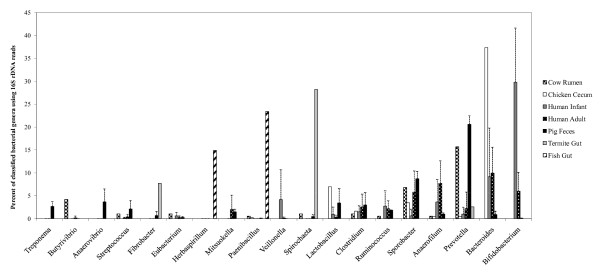
**Taxonomic distribution of bacterial genera from swine and other currently available gut microbiomes within MG-RAST**. The percent of sequences assigned to each bacterial order from swine and other gut metagenomes is shown. Using the "Phylogenetic Analysis" tool within MG-RAST, each gut metagenome was searched against the RDP and greengenes databases using the BLASTn algorithm. The percentage of each bacterial phlya from swine, human infant, and human adult metagenomes were each averaged since there was more than one metagenome for each of these hosts within the MG-RAST database. The e-value cutoff for 16S rRNA gene hits to the RDP and greengenes databases was 1×10^-5 ^with a minimum alignment length of 50 bp.

**Figure 4:**
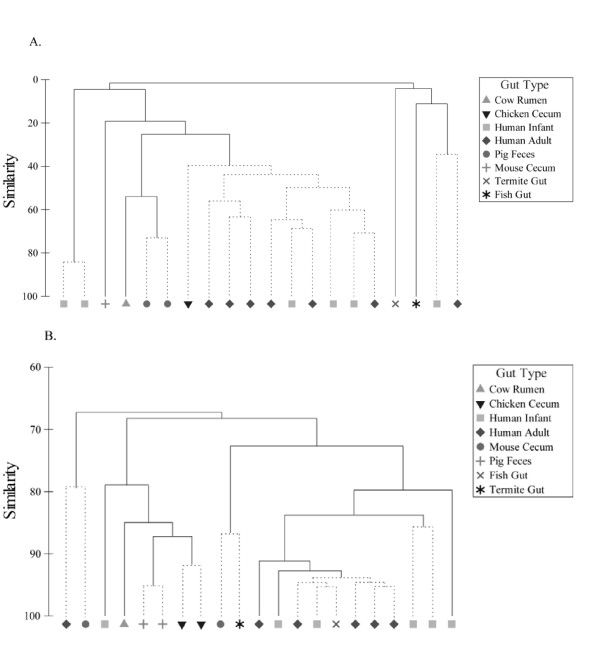
**Hierarchical clustering of gut metagenomes available within MG-RAST based on the taxonomic (A) and functional (B) composition**. A matrix consisting of the number of reads assigned to the RDP database was generated using the "Phylogenetic Analysis" tool within MG-RAST, using the BLASTn algorithm. The e-value cutoff for 16S rRNA gene hits to the RDP database was 1×10^-5 ^with a minimum alignment length of 50 bp. A matrix consisting of the number of reads assigned to SEED Subsytems from each gut metagenome was generated using the "Metabolic Analysis" tool within MG-RAST. The e-value cutoff for metagenomic sequence matches to this SEED Subsystem was 1×10^-5 ^with a minimum alignment length of 30 bp. Resemblance matrices were calculated using Bray-Curtis dissimilarities within PRIMER v6 software [38]. Clustering was performed using the complete linkage algorithm. Dotted branches denote that no statistical difference in similarity profiles could be identified for these respective nodes, using the SIMPROF test within PRMERv6 software.

Among the Bacteroidetes, *Prevotella *were significantly more abundant in the swine fecal metagenome when compared to all other gut metagenomes (p < 0.05), with the exception of the cow rumen, while *Bacteroides *species were more abundant in chicken and human distal gut microbiomes (Figure 3). Additionally, *Anaerovibrio *and *Treponema *genera were exclusively found within the pig fecal metagenomes. Hierarchical clustering of phylotype distribution (genus-level) from each gut microbiome revealed that community structure of the swine fecal microbiome was significantly different (p < 0.05) from the other gut microbiomes (Figure 4A). Of all the microbiomes used in the comparative analysis, the swine metagenomes exhibited the highest resemblance to the cow rumen, displaying 59% similarity at the genus level. Surprisingly, swine fecal community structure (genus-level) was less than 40% similar to any of the human fecal microbiomes used in this study.

### Diversity of swine gut microbiome

In order to assess diversity of each gut metagenome, several statistical models were applied for measuring genotype richness, evenness, and coverage of rRNA gene hits against the RDP database. Overall, while coverage of the GS20 pig fecal metagenome was slightly lower than the FLX run (91% vs 97%), all diversity indices showed that both swine metagenomes had similar genotype diversity (Table 2). Swine fecal microbiomes appeared to have higher richness and lower evenness as compared to chicken, mouse, fish, and termite gut communities. This trend was further supported by a cumulative k-dominance plot, as both swine k-dominance curves are less elevated than all other gut microbiomes (Additional File 1, Fig. S4). Rarefaction of the observed number of OTUs (genus-level) indicated several of the individual human microbiomes were under-sampled (Additional File 1, Fig. S5), thus, we combined individual pig fecal, human infant, and human adult rRNA gene hits, and also performed diversity analyses on the total number of rRNA gene hits (Table 2). While the number of rRNA gene sequences in metagenome projects is low, comparison between available metagenomes showed that the human adult and pig microbiomes shared similar diversity patterns, and were more diverse than human infant microbiota.

**Table 2:** Diversity analyses of the gut microbiomes using 16S rRNA gene sequences

Metagenome	Sobs	Chao1	ACE	Jackknife	Shannon	Shannon (non-parametric)	Simpson	boneh	coverage
**Pig Feces GS20**	52	77.09 (61.24-120.12)	116.05 (89.07-162.68)	76.88 (62.74-91.02)	3.17 (3.03-3.32)	3.36	0.07 (0.05-0.08)	10.34	0.91
**Pig Feces FLX**	71	113.86 (86.42-190.10)	125.60 (103.78-161.95)	119.78 (92.49-147.06)	3.19 (3.10-3.29)	3.27	0.08 (0.07-0.09)	5.84	0.97
**Cow Rumen**	40	63.00 (48.33-103.51)	168.17 (120.97-242.89)	63.63 (49.92-77.33)	2.56 (2.35-2.77)	2.86	0.15 (0.11-0.19)	10.58	0.88
**Chicken Cecum**	37	47.11 (39.89-72.43)	68.02 (52.45-99.29)	51.00 (40.63-61.37)	2.25 (2.11-2.39)	2.36	0.20 (0.17-0.23)	5.58	0.97
**Human In-A**	20	33.75 (23.40-75.55)	62.23 (41.01-104.88)	32.94 (22.19-43.70)	2.52 (2.25-2.79)	2.84	0.10 (0.06-0.15)	5.05	0.81
**Human In-B**	10	20.50 (12.03-64.19)	27.79 (13.32-105.26)	23.03 (10.30-35.76)	0.84 (0.50-1.17)	1.15	0.68 (0.53-0.82)	3.02	0.90
**Human In-D**	26	32.00 (27.33-53.10)	34.06 (28.41-52.93)	35.00 (26.68-43.32)	2.97 (2.80-3.13)	3.16	0.05 (0.04-0.07)	4.95	0.90
**Human In-E**	18	22.20 (18.79-40.34)	26.41 (20.24-49.62)	25.00 (17.67-32.33)	1.11 (0.88-1.34)	1.26	0.60 (0.51-0.69)	3.72	0.96
**Human In-M**	26	46.00 (32.02-92.48)	80.76 (54.86-129.91)	43.95 (31.51-56.39)	2.97 (2.72-3.22)	3.42	0.05 (0.02-0.08)	7.34	0.69
**Human In-R**	21	23.50 (21.41-36.27)	26.77 (22.44-44.13)	27.00 (20.21-33.79)	2.57 (2.38-2.76)	2.72	0.10 (0.07-0.13)	2.83	0.87
**Human F1-S**	22	31.00 (24.00-62.45)	39.21 (29.33-62.40)	31.00 (22.68-39.32)	2.68 (2.49-2.87)	2.85	0.08 (0.06-0.10)	4.30	0.90
**Human F1-T**	37	64.14 (46.04-118.51)	109.84 (79.72-161.17)	66.22 (47.95-84.48)	3.05 (2.83-3.26)	3.36	0.07 (0.04-0.10)	9.39	0.82
**Human F1-U**	17	20.75 (17.64-39.02)	21.96 (18.14-38.53)	23.00 (16.21-29.79)	2.30 (2.04-2.56)	2.49	0.15 (0.08-0.21)	3.22	0.91
**Human F2-V**	37	46.10 (39.59-68.96)	48.59 (41.00-70.52)	51.00 (40.63-61.37)	3.07 (2.89-3.26)	3.29	0.07 (0.05-0.09)	7.64	0.87
**Human F2-W**	25	36.00 (27.88-66.94)	55.50 (39.11-90.92)	37.00 (27.40-46.60)	2.72 (2.50-2.93)	2.96	0.08 (0.06-0.11)	5.85	0.86
**Human F2-X**	19	21.00 (19.29-32.96)	22.80 (19.83-36.32)	24.00 (17.80-30.20)	2.57 (2.38-2.76)	2.72	0.09 (0.06-0.12)	3.06	0.94
**Human F2-Y**	27	40.20 (30.44-77.60)	41.54 (31.66-72.36)	39.78 (29.54-50.01)	2.87 (2.67-3.08)	3.10	0.07 (0.05-0.09)	5.82	0.87
**Mouse Cecum**	14	36.50 (19.23-110.77)	41.22 (20.35-130.67)	39.09 (19.22-58.95)	2.18 (1.78-2.58)	2.69	0.15 (0.04-0.25)	4.13	0.67
**Termite Gut**	13	27.00 (15.92-80.11)	30.75 (16.84-95.03)	29.19 (14.56-43.82)	2.05 (1.72-2.38)	2.38	0.16 (0.09-0.23)	3.39	0.79
**Fish gut**	14	19.00 (14.86-42.91)	20.45 (15.44-42.93)	20.00 (13.21-26.79)	2.29 (2.05-2.54)	2.50	0.11 (0.07-0.15)	3.71	0.87
**Pig Feces Total**	91	127.25 (105.56-181.27)	184.42 (150.70-237.20)	127.57 (108.75-146.39)	3.15 (3.11-3.20)	3.19	0.06 (0.06-0.07)	0.34	0.99
**Human Infant Total**	59	80.00 (66.47-118.05)	83.37 (69.43-115.92)	82.03 (68.30-95.75)	2.66 (2.52-2.79)	2.78	0.17 (0.14-0.20)	1.25	0.96
**Human Adult Total**	72	89.00 (77.34-126.16)	85.74 (77.28-107.71)	89.60 (77.72-101.48)	3.35 (3.30-3.40)	3.39	0.05 (0.04-0.05)	0.37	0.99

### Functional classification of the swine gut metagenome

To predict the metabolic potential within the swine fecal microbiome, both the MG-RAST and the IMG/M-ER annotation pipelines were used. The broad functional classifications of the swine fecal metagenomic reads were expected from previous metagenomic studies of the chicken cecum, cow rumen, human distal gut, and the termite gut. Similar proportions of broad level SEED subsystem classification were retrieved for both the GS20 and FLX swine fecal metagenomes (Additional File 1, Fig. S6). However, only 10% of sequences retrieved from the GS20 pig fecal metagenome were assigned to 574 subsystems, while more than 25% of all FLX reads were classified into 714 subsystems. This is compatible with the longer reads produced by the latter instrument, which allows for more robust gene predictions. When both pig fecal metagenomes were annotated using proxygenes within the JGI IMG/M ER pipeline, nearly one third of all GS20 and FLX pig fecal metagenomes were assigned to Pfams, and over 20% were assigned to COGs. This finding suggests that the proxygene method for gene-centric approaches to metagenomic studies is more robust than the direct BLASTx assignment strategy. Diversity analyses of Subsystems, COGs, and Pfams retrieved from swine metagenomes and other gut metagenomes tested in this study, revealed that larger sequencing efforts generate significantly more functional classes (Additional File 2, Tables S4 & S5). For example, an additional 150 Subsystems, 896 COGs, and 1271 Pfams were retrieved from the FLX run as compared to the GS20 metagenome, suggesting additional sequencing efforts for all gut microbiomes are necessary to cover the high functional diversity in gut environments.

Carbohydrate metabolism was the most abundant SEED subsystem (MG-RAST annotation pipeline) representing 13% of both swine fecal metagenomes (Additional File 1, Fig. S6). Genes associated with cell wall and capsule, stress, and virulence were also very abundant in both metagenomes. Approximately 16% of annotated reads from swine fecal metagenomes were categorized within the clustering-based subsystems, most of which have unknown or putative functions. Additionally, 75% to 90% of metagenomic reads were not assigned to subsystems, suggesting the need for improved binning and coding region prediction algorithms to annotate these unknown sequences.

To improve the meaning of metagenomic functional analysis, we applied statistical methods to compare the 29 broad level functional subsystems that are more or less represented in the different microbiomes. As was expected, all gut metagenomes were dominated by carbohydrate metabolism subsystems with amino acid, protein, cell wall and capsule, and virulence subsystems represented in relatively high abundance as well. Protein metabolism and amino acid subsystems were significantly more abundant in chicken, pig, and cow gut metagenomes (Additional File 1, Fig. S7). Additionally, the termite, fish, and pig gut had a higher proportion of reads classified to the chemotaxis and motility subsystems as compared to other gut metagenomes.

### Comparative gut metagenomics

In this study, we examined the functional similarity of the Yorkshire pig fecal metagenome by comparing it to currently available metagenomic projects. Hierarchical clustering of functional profiles derived from gut metagenomes available in the MG-RAST database revealed that the GS20 and FLX swine fecal datasets shared approximately 70% similarity to other human metagenomes (Figure 4B). This analysis also showed the swine gut metagenome clustered more closely with chicken cecal and cow rumen metagenomes than to the human gut metagenomes (Figure 4B).

We further investigated subsystems associated with specialized cell wall and capsule enzymes, DNA recombination, and prophage genes since they were very abundant in the swine fecal metagenome (Additional File 1, Fig. S8). Within the DNA recombination and prophage subsystem, the swine fecal metagenome was enriched for RstA phage-related replication proteins, terminases, and portal proteins. Additionally, more than 30 metagenomic contigs (i.e., > 500 bp) shared high homology to unknown phage proteins. For proteins involved in the cell wall and capsule subsystem, unknown glycosyl transferases, a phosphoglucosamine mutase, and a phosphotransferase were over abundant in the swine metagenome (Table 3). N-acetyl glucosamine-specific PTS system, proteins involved in mannose uptake, and novel capsular polysaccharide synthesis enzymes were exclusively found within the swine fecal metagenome. Hierarchical clustering of all genes retrieved from the cell wall and capsule functional subsystem for each gut microbiome revealed that swine fecal cell wall/capsule profiles were greater than 60% similar to those of the cow rumen. Additionally, cell wall and capsule profiles in the swine samples were more similar to termite gut than the human gut (Additional File 1, Fig. S9). When carbohydrate subsystems were compared across gut microbiomes, maltose and maltodextrin utilization were the most abundant carbohydrate subsystem in the swine, termite, and cow rumen. Analysis of carbohydrate metabolism using the SEED subsystem approach, revealed several proteins unique to the swine gut metagenome such as an outer surface protein part of the cellobiose operon, a beta-glucoside-specific IIA component and a cellobiose-specific IIC component of the PTS system, and a protein similar CDP-glucose 4,6-dehydratase.

**Table 3:** List of cell wall and capsule SEED subsystem functions overabundant in swine fecal metagenome

	Pig Feces	Human Adult	Human Infant	Cow Rumen	Termite Gut	Mouse Cecum	Fish gut
**putative glycosyltransferase - possibly involved in cell wall localization and side chain formation of rhamnose-glucose polysaccharide**	112	9	10	10	0	1	0
**Phosphoglucosamine mutase (EC 5.4.2.10)**	97	18	9	0	20	0	1
**COG3178: Predicted phosphotransferase related to Ser/Thr protein kinases**	66	10	6	4	5	2	1
**3-deoxy-D-manno-octulosonate 8-phosphate phosphatase (EC 3.1.3.45)**	27	10	9	2	0	1	3
**O-antigen export system, permease protein**	23	3	2	4	0	0	1
**Glutamine synthetase, clostridia type (EC 6.3.1.2)**	21	4	1	3	0	0	0
**D-glycero-D-manno-heptose 1-phosphate guanosyltransferase**	20	7	6	1	0	5	0
**UDP-glucose 4-epimerase (EC 5.1.3.2)**	14	1	2	0	9	1	1
**Capsular polysaccharide synthesis enzyme Cap8D**	9	0	1	1	0	0	0
**D-alanine--D-alanine ligase B (EC 6.3.2.4)**	8	0	0	0	0	0	0
**PTS system, N-acetylglucosamine-specific IIB component (EC 2.7.1.69)**	7	0	0	0	0	0	0
**Mannose-1-phosphate guanylyltransferase (GDP) (EC 2.7.7.22)**	5	0	0	0	0	0	0
**2-Keto-3-deoxy-D-manno-octulosonate-8-phosphate synthase (EC 2.5.1.55)**	3	0	0	0	0	0	0
**capsular polysaccharide biosynthesis protein, putative**	3	0	0	0	0	0	0
**Capsular polysaccharide synthesis enzyme Cap8L**	3	0	0	0	0	0	0

**Figure 5:**
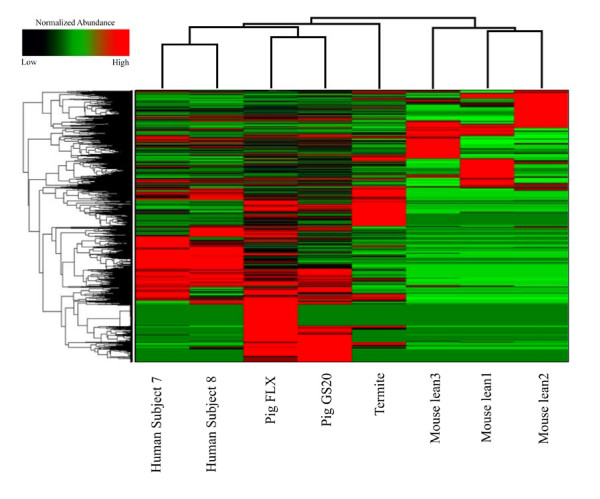
**Two-way hierarchical clustering of functional gene groups from swine and other currently available gut metagenomes within JGI's IMG/M database**. Hierarchical clustering was performed using a matrix of the number of reads assigned to COGs from each gut metagenome, which was generated using the "Compare Genomes" tool in IMG/M ER. COGs less abundant in a given metagenome are shown in black/darkgreen, while more abundant COGs are shown in red.

Two-way hierarchical clustering of COGs retrieved from swine, human, termite, and mouse gut microbiomes revealed several suites of gene families unique to the swine distal gut (Figure 5). Additionally, the swine fecal FLX run yielded a pool COGs unique to the FLX run, suggesting the deeper level of sequencing uncovered a larger proportion of functional diversity. Interestingly, this analysis unveiled a large collection of COGs unique to the swine fecal metagenome.

## Discussion

The primary goal of this study was to characterize the functional content of the swine fecal microbiome. We also compared the pig distal gut samples to other currently available gut metagenomes, as a method for revealing potential differences in gut microbial systems. The comparative metagenomic approach used in this study identified unique and/or overabundant taxonomic and functional elements within the swine distal gut. It also appears that the genes associated with the variable portion of gut microbiomes cluster by host environment with surprising hierarchical trends. Thus, our findings suggest that while a majority of metagenomic reads were associated with a relatively conserved core microbiome, the variable microbiome carries out many unique functions [8]. The data also suggest that taxonomically diverse gut organisms maintain a conserved core set of genes, although it should be noted that the variable microbiome is more abundant than previously anticipated. For example, of the 160 functional SEED Subsystems, DNA repair/recombination subsystems were amongst the most abundant functions within all gut microbiomes. Since the frequency of a gene encoding a particular metabolic function is usually related to its relative importance in an environment [8], transferable elements are likely to be very important in shaping microbiome composition and diversity in gastrointestinal environments. When comparing prophage and transposon genes from each gut microbiome, the pig distal microbiome examined in this study harbored an abundant and diverse array of horizontal gene transfer mechanisms. When putative transposases for all available gut metagenomes were retrieved using the IMG/M annotation pipeline, the swine fecal metagenome harbored the most diverse transposase profiles (i.e., 26 different transposase families; Additional File 1, Fig. S10). The potential importance of transposable elements was further supported by the fact that 42% of large contigs (> 500 bp) assembled from all pig fecal metagenomic contained sequences that matched putative transposases (Table 4). Additionally, 24% of all large contigs matched to proteins associated with antibiotic resistance mechanisms. These results suggest that lateral gene transfer and mobile elements allow gut microbial populations to perpetually change their cell surface for sensing their environment and collecting nutrient resources present in the distal intestine [2].

**Table 4:** Summary of BLASTX results of pig fecal assembled contigs

Contig Name	Contig Length	Number of Reads	Predicted Protein	Organism	Accession Number	E-value	Percent Identity
**Contig09884**	1444	159	hypothetical protein	*Bacteroides fragilis*	BAA95637	0	99%
**Contig00095**	646	22	tetracycline resistant protein TetQ	*Bacteroides *sp. D1	ZP 04543830	2.00E-111	99%
**Contig01271**	812	22	tetracycline resistance protein	*Prevotella intermedia*	AAB51122	3.00E-102	98%
**Contig01956**	731	17	macrolide-efflux protein	*Faecalibacterium prausnitzii *A2-165	ZP 05613628	3.00E-85	99%
**Contig01189**	549	14	macrolide-efflux protein	*Bacteroides *finegoldii DSM 17565	ZP 05859238	8.00E-83	98%
**Contig00070**	603	11	rRNA (guanine-N1-)-methyltransferase	*Faecalibacterium prausnitzii *A2-165	ZP 05614052	2.00E-81	100%
**Contig07794**	846	27	putative transposase	*Bacteroides fragilis*	AAA22911	4.00E-81	98%
**Contig03360**	671	10	ABC transporter, ATP-binding protein	*Bacillus thuringiensis *serovar pondicheriensis BGSC 4BA1	ZP 04090641	8.00E-77	77%
**Contig09748**	650	13	hypothetical protein PRABACTJOHN 03572	*Parabacteroides johnsonii *DSM 18315	ZP 03477882	9.00E-71	77%
**Contig00180**	846	26	macrolide-efflux protein	*Faecalibacterium prausnitzii *A2-165	ZP 05613628	6.00E-67	90%
**Contig00608**	527	7	ISPg3, transposase	*Prevotella *tannerae ATCC 51259	ZP 05734821	1.00E-59	67%
**Contig04843**	578	7	hypothetical protein COPEUT 02459	*Coprococcus eutactus *ATCC 27759	ZP 02207638	2.00E-57	88%
**Contig00340**	847	24	conserved hypothetical protein	*Bacteroides *sp. 4 3 47FAA	ZP 05257903	6.00E-56	72%
**Contig02245**	616	7	putative transposase	*Bacteroides *thetaiotaomicron VPI-5482	NP 809147	3.00E-52	62%
**Contig09776**	531	9	resolvase, N domain protein	*Faecalibacterium prausnitzii *A2-165	ZP 05613620	5.00E-41	100%
**Contig02310**	557	11	replication initiator protein A	*Faecalibacterium prausnitzii *A2-165	ZP 05613624	1.00E-38	100%
**Contig02075**	524	9	Transposase	*Bacteroides fragilis *3 1 12	ZP 05284372	7.00E-38	92%
**Contig02837**	529	7	hypothetical protein CLOSS21 01510	*Clostridium *sp. SS2/1	ZP 02439046	6.00E-37	67%
**Contig09732**	632	11	hypothetical protein BACCOP 00975	*Bacteroides *coprocola DSM 17136	ZP 03009123	1.00E-35	62%
**Contig09862**	574	16	conserved hypothetical protein	*Oxalobacter formigenes *HOxBLS	ZP 04576182	1.00E-34	100%
**Contig00069**	897	21	regulatory protein	*Sphingobacterium spiritivorum *ATCC 33300	ZP 03965851	4.00E-29	43%
**Contig00129**	529	9	transposase, putative	*Bacteroides *sp. 2 1 7	ZP 05288481	8.00E-26	75%
**Contig00130**	674	11	hypothetical protein BACCOP 00975	*Bacteroides *coprocola DSM 17136	ZP 03009123	6.00E-24	43%
**Contig09924**	1355	55	conserved hypothetical protein	*Magnetospirillum gryphiswaldense *MSR-1	CAJ30045	2.00E-23	45%
**Contig00140**	552	13	ISPg7, transposase	*Cyanothece *sp. PCC 8802	YP 003135760	5.00E-23	44%
**Contig00572**	675	16	transposase, putative	*Bacteroides *sp. 2 1 7	ZP 05288481	2.00E-21	57%
**Contig09792**	556	9	hypothetical protein ALIPUT 01364	*Alistipes putredinis *DSM 17216	ZP 02425220	2.00E-16	67%
**Contig09902**	528	14	putative transposase	*Lentisphaera araneosa *HTCC2155	ZP 01873850	2.00E-12	63%
**Contig09796**	867	17	hypothetical protein CLONEX 03424	*Clostridium *nexile DSM 1787	ZP 03291203	3.00E-07	35%
**Contig01049**	548	5	No significant similarity found	-	-	-	-
**Contig04775**	565	4	No significant similarity found	-	-	-	-
**Contig09740**	531	7	No significant similarity found	-	-	-	-
**Contig09927**	656	29	No significant similarity found	-	-	-	-

Interestingly, a majority of these transposable elements belonged to the Bacteroidetes genomes. These genetic elements have been shown to aid in the adaptation of this diverse group of bacteria to the distal gut environments [2]. Many of the genetic features unique to the swine fecal metagenome encoded cell surface features of different Bacteroidetes populations, suggesting the adaptation of Bacteroidetes populations to distinct niches within the swine distal gut microbiome. While the precise role of diet, antibiotic usage, and genetics on shaping the ecology of the distal pig gut will require further study, it should be noted that industrialization of the swine industry has lead to the frequent use antibiotics to supplement the pig diet to maintain and increase meat production.

Studying the swine distal gut metagenome also shed light on the diversity and high occurrence of antibiotic resistance mechanisms employed by the microbiome (Additional File 1, Fig. S11). Antibiotics are widely used as additives in food or water within swine feeding operations to prevent and treat animal disease and to promote animal growth [19]. Seepage and runoff of swine waste into both surface and groundwater with antibiotics and antibiotic-resistant bacteria poses a significant threat to public health. Nearly 6% of all assigned metagenomic reads retrieved from both swine fecal metagenomes were involved in antibiotic resistance mechanisms. Interestingly, tetracycline resistance was the most abundant class of virulence subsystems within the swine fecal metagenome, which may be explained by the fact that this antibiotic class was used in the diet supplied to the animals associated with this study. This antibiotic class is reported as comprising nearly half of the total amount of antibiotics used in commercial swine operations [20].

Resistance to fluoroquinolones was also well represented in the swine fecal metagenome, and may be explained by the increase of its non-therapeutic use within pig feed. While early studies indicated there was a low risk of fluoroquinolone resistance, recent studies are showing the use of fluoroquinolones is among the most important factors associated with finding resistant *E. coli *and *Campylobacter *in animal operations [21]. Interestingly, there was no history of fluoroquinolone use on the swine farm from which these samples were collected. Fluoroquinolone resistance has been found on farms with no history of fluoroquinolone use, suggesting that resistant organisms, such as *Campylobacter *have the ability to spread between pig farms. Genes with high sequence similarity to methicillin-resistant *Staphylococcus *subsystem were also retrieved in this study. This finding is important considering MRSA carriage has been elevated in swine and exposed farmers and veterinarians [22], suggesting that MRSA infection is a significant risk in swine farm resident and worker cohorts.

More than 12% of virulence subsystems identified in the pig fecal metagenome were classified as multi-drug resistance mechanisms, suggesting the pig gut could be a hot-spot for multiple-antibiotic resistant bacteria. One subsystem, the MexA-MexB-OprM multiple drug efflux pump was found exclusively in the swine fecal metagenome. This antibiotic resistance mechanism has been detected only in *Pseudomonas aeruginosa *strains known to carry resistance in cystic fibrosis patients [23] and has not been previously described in distal gut environments. Additionally, more than 10% of virulence-associated sequences were assigned to yet-to-be-described virulence subsystems, suggesting that unknown virulence mechanisms are at work within the distal gut. Altogether, the high abundance of metagenomic sequences assigned to known and unknown antibiotic resistance subsystems suggests that functional metagenomics is an adequate tool for assessing the prevalence of antibiotic resistance within high cell density environments.

**Figure 6:**
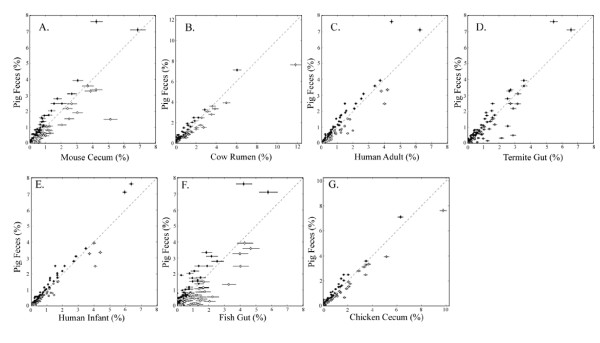
**Pair-wise comparisons of functional gene groups from swine versus other gut metagenomes**. Pair-wise comparisons were calculated for the pig fecal metagenome versus (A) lean mouse cecum (B) cow rumen (C) human adult (D) termite gut (E) human infant (F) fish gut (G) and chicken cecal metagenomes is shown. Each point on this exploratory plot represents a different SEED Subsystem and it's relative abundance within the pig fecal metagenome compared to other available gut metagenomes within the MG-RAST database. Points closer to y-axis represent functions more abundant in the swine gut metagenome, while points closer to the x-axis are more abundant in other gut metagenomes. Points laying on or near the dotted midline have equal or very similar abundances within both metagenomes. A matrix of the abundance of sequences assigned to each SEED Subsystem from each gut metagenome was generated using the "Metabolic Analysis" tool in MG-RAST. The number of reads from each individual pig, human infant, and human adult metagenomes were each combined since there was more than one metagenome for each of these hosts within the MG-RAST database. The e-value cutoff for metagenomic sequence matches to SEED Subsystems was 1×10^-5 ^with a minimum alignment length of 30 bp. Fisher exact tests were used with the Benjamin-Hochberg FDR multiple test correction to generate a list of significantly different SEED Subsystems using STAMP v1.0.2 software [39]. The Newcombe-Wilson method was used to calculate the 95% confidence intervals.

Pair-wise comparisons of each gut metagenome (MG-RAST SEED database) with the swine gut revealed 15 SEED subsystems that were significantly different in abundance for the swine fecal metagenome (Figure 6 and Additional File 1, Fig. S12). Two subsystems in particular were statistically significantly overabundant in the swine gut metagenome, as compared to all other gut metagenomes: the UDP-N-acetylmuramate from fructose-6-phosphate biosynthesis and folate biosynthesis. UDP-N-acetylmuramate is a peptidoglycan-derived muropeptide that as a group are considered to be potential virulence factors of several gut pathogens [24] specifically involved in biofilm colonization. Higher abundances of genes related to folate biosynthesis may be a direct result of supplemental amounts of folic acid in swine feedstuff or an increased production by the swine microbial consortia [25]. The impacts of food additives, such as folic acid, on the microbial ecology of the swine gut warrants further study.

Comparative metagenomics of proteins involved in the cell wall and capsule subsystems revealed several unique glycosyl transferases and carbohydrate uptake systems. This unique pool of glycosyl transferases may provide a capacity for diversification of surface polysaccharide structures helping shape the genetic functional potential of this gut ecosystem. For example, the acquisition of new types of carbohydrate-binding proteins, transporters, and degradation enzymes through horizontal gene transfer may allow for the utilization of a wider array of substrates that may be utilized for energy harvesting [2]. Pfams and COGs related to virulence factors such as adhesions were numerous within the gene families unique to the swine fecal metagenomes (Additional File 2, Table S6). Proteins involved in carbohydrate transport and attachment were both abundant and unique to the distal swine gut with more than 50 metagenomic contigs sharing high sequence homology to putative carbohydrate membrane transporters. Other proteins involved in carbohydrate metabolism were unique to the swine metagenome including glycosyl hydrolases, cellobiohydrolases, gluconolactonases, maltodextrin metabolism, and pectin lyases. The identification of unique gene families provides one line of evidence that the variable microbiome is a result of the microbial interaction with its surrounding environment. Because the environment surrounding gut microbes can vary among host species, a direct result of this level of functional diversity may be the generation of swine-specific microbiomes. Many proteins of unknown functions were also unique to the swine fecal metagenome, suggesting that some of them may be engaged in novel functions that have important biological meaning.

The high functional similarity between the pig and human metagenome is not surprising in light of the fact that they are mammalian omnivores with similar digestive tract structures and functions. Results from 16S rRNA gene sequence analyses suggest that bacterial gut communities are similar among omnivorous mammals [2]. Similarities at the phylogenetic level between pig and human guts include the large presence of Firmicutes and members of the Bacteroidetes as the most abundant Gram-negative bacteria in their gastrointestinal tracts [14]. While differences in the relative abundance of *Lactobacilli *phylotypes have been noted, our data provides for the first time a functional perspective on how similar pigs and humans gut systems in spite of the differences in microbial community structure. In contrast, the functional similarities shared between the swine fecal metagenome and the termite gut was surprising and suggestive of previously unknown shared metabolic capabilities between these gut environments. For example, the pig and termite were the only two hosts possessing a suite of functions involved in archaeal lipid biosynthesis (Additional File 2, Fig. S13), suggesting an intimate relationship between the swine and archaeal gut populations [26]. Swine-specific methanogenic populations have been demonstrated in previous studies [17,27]. Similarities in cell wall and capsule profiles between the swine samples and termite gut may indicate that these functions can endow the swine gut with diversification of surface polysaccharide structures, allowing the host immune system to accommodate a diverse microbiota [2]. Presence of novel carbohydrate binding proteins and transporters also suggest the swine gut is capable of exploiting a diverse array of substrates.

Similarities in functional gene profiles (SEED subsystem abundance) among swine, chicken cecal and cow rumen metagenomes as compared to human gut metagenomes were unexpected considering the similarity shared between pig and human gut anatomy and physiology. These results suggest that that some microbial functions within the swine gut are shared among other agricultural animals, with arguably very different gastrointestinal anatomy and physiologies. For example, the elevated abundance of genes associated with protein turnover in pigs, chicken, and cow gut metagenomes is consistent with an increased use of amino acids for protein accretion in food production animals and is also consistent with the high protein diet fed to the pigs in this study. Additionally, the high abundance and diversity of carbohydrate utilization subsystems found in this swine metagenome may be a result of the high level of complex polysaccharides found in the diet. Altogether these data suggest that agricultural animal husbandry practices can impose significant selective pressures on the gut microbiota, regardless of gut type.

Surprisingly, this pig fecal metagenome revealed the presence of motile *Treponema *and *Anaerovibrio *genera. The presence of sequences associated with *Treponema *in this study (i.e., 3-4% of all sequences swine fecal metagenome) suggests an order of magnitude higher abundance than a previous study in which swine gut microbiota revealed a very low abundance of Spirochetes using a culture independent method (i.e., 0.3% of all phylotypes) [14]. This genus has been previously detected in swine colonic samples but their presence in elevated levels is normally associated with swine dysentery. Discrepancies in community composition between cloning-based methods and non-cloning based methods have been reported in the literature, primarily attributed to PCR amplification biases [28,29]. While many mammalian gut microbial communities are dominated by non-motile microbes, the termite hindgut and the fish gut harbor motile populations of bacteria, which are known to possess complex social behaviors [12,30,31]. This study revealed the pig gut may harbor previously unknown social dynamics, which may be relevant for maintaining compartmentalization and promoting niche selection within monogastric systems.

## Conclusions

Herein, we report the first shotgun metagenomic pyrosequencing approach to study the microbiome of the swine distal gut. The overall goal of this study was to characterize the swine fecal microbiome with respect to species composition and functional content. Comparative metagenomic analyses identified unique and/or overabundant taxonomic and functional elements within swine distal gut microbiomes. These genetic attributes may help us better understand the microbial genetic factors that are relevant to swine health. Genes associated with the variable portion of gut microbiomes clustered by host environment with surprising hierarchical trends, suggesting that the variable microbiome content of a given host species may be reflective of the host ecology. While a larger metagenomic database that includes information on intra-host variation is needed for swine and other gut systems, this study provides a baseline for understanding the complexity of the swine gut microbial ecology, while also highlighting striking similarities and differences when compared to other animal gastrointestinal environments.

## Methods

### Fecal Sample Collection

Fecal samples were collected from eight, six-month old Yorkshire pigs from a large swine operation located in Northeastern Ohio, which housed more than 1,000 head of swine at the time of collection. Swine were weaned eight weeks after birth. Their diets consisted of a high-energy corn-soybean meal diet containing 14.00% crude protein, 0.63% lysine, 3.00% crude fat, 4.00% crude fiber, 0.55%- 0.70% calcium, 0.52% phosphorus, 0.35%-0.50% salt, 0.3 ppm selenium, 80 ppm zinc. (Kalmbach Feeds, OH). In addition, swine were supplemented with feed grade antibiotics for improvement in growth performance. Antibiotics consisted of chlortetracycline and penicillin at the concentration of 20 g per ton of feed. Fecal samples were transported to the laboratory on ice within four hours of collection, and stored at -20°C until further processing. Fecal DNA was extracted with the FastDNA SPIN Kit (MP Biomedicals, Inc., Solon, OH) according to the manufacturer's instructions using 0.25 g of each fecal sample. Total DNA was quantified using a NanoDrop^® ^ND-1000 UV spectrophotometer (NanoDrop Technologies, Wilmington, DE).

### Pyrosequencing and Gene Annotation

A total of 24 μg (3 μg of each fecal DNA extract, n = 8) were pooled and sent for pyrosequencing to 454 Life Sciences, where two different sequencing runs were performed. The first run was performed using Genome Sequencer GS20 platform while the Genome Sequencer FLX instrument was used for the second run. Each pig fecal metagenomic sequencing run was assembled *de novo *using the Newbler assembly software by 454 Life Sciences. The metagenomes used in this paper are freely available from the SEED, JGI's IMG/M, and NCBI Short Read Archive. The NCBI genome project ID and GOLD ID for swine fecal GS20 and FLX metagenomic sequencing runs generated in this project are 39267 and Gm00197, respectively.

Raw sequencing reads from both datasets were submitted to the Joint Genome Institute's IMG/M-ER annotation pipeline using the proxygene method for gene annotation [4,32]. Additionally, both metagenome runs were annotated using the "Phylogenetic Analysis" tool within the MG-RAST pipeline [33]. The BLASTn algorithm (e-value less than1 × 10^-5 ^and a sequence match length greater than 50 nucleotides) was used to identify small subunit rRNA genes from RDP [34], SILVA SSU [35], and Greengenes databases [36]. Within the MG-RAST pipeline, the "Metabolic Analysis" tool was used to search sequences from pig fecal metagenomes against the SEED database using the BLASTx algorithm (e-value less than 1×10^-5 ^and a sequence match length greater than 30 nucleotides) [37].

### Comparative Metagenomics and Statistical Analyses

Comparative metagenomics was performed using both the IMG/M and MG-RAST pipelines. GS20 and FLX pig metagenomic runs were compared to the current publicly available gut metagenomes within each of these databases. Within the IMG/M pipeline, the two pig metagenomic runs were compared against three lean mouse (*Mus musculus strain *C57BL/6J) cecal metagenomes (Metagenome names: Mouse Gut Community lean1-3), two healthy human fecal metagenomes (Metagenome names: Human Gut Community Subject 7-8), and one termite (*Nasutitermes sp*) hindgut metagenome (Metagenome name: Termite hindgut). Descriptive information about these mouse, human and termite metagenomes can be found in the GOLD database under Gm00071, Gm00052, Gm00013 GOLD IDs, respectively. Within IMG/M the "Compare Genomes" tool was chosen to extract COG and Pfam protein profiles from the swine, mouse, human, and termite gut microbiomes. These profiles were then normalized for sequencing coverage by calculating the percent distribution, prior to downstream statistical analysis. To find over-abundant or unique functions to a given metagenomic dataset, a two-way hierarchical clustering of normalized COG and Pfam abundances was performed using the Bioinformatics Toolbox with Matlab version 2009a. Additionally, to determine if unique or overabundant functions were statistically meaningful, the binomial test within the Shotgun FunctionalizeR program was employed [38].

The GS20 and FLX pig fecal datasets were also compared against gut metagenomes available within the MG-RAST metagenomic annotation pipeline. The two pig fecal metagnonomic datasets were compared against the following MG-RAST metagenomic projects: cow rumen (Cow Rumen Project: 444168.3), chicken cecum (FS-CAP Project:4440285.3), human infant subjects In-A, In-B, In-D, In-E, In-M and In-R (Human Faeces Projects: 4440946.3, 4440945.3, 4440948.3, 4440950.3, 4440949.3, 4440951.3), human adult subjects F1-S, F1-T, F1-U, F2-V, F2-W, F2-X, and F2-Y (Human Faeces Projects: 4440939.9, 4440941.3, 4440940.3, 4440942.3, 4440943.3, 4440944.3, and 4440947.3), healthy fish gut (Fish Gut Project: 4441695.3), and lean mouse cecum (Human Faeces Project: 4440463.3). Within MG-RAST, phylogenetic information was extracted from these gut metagenomes using RDP [31], SILVA SSU [32], and Greengenes[33] databases (e-value less than 1 × 10^-5 ^and a sequence match length greater than 50 nucleotides). These taxonomic profiles were then normalized for differences in sequencing coverage by calculating percent distribution, prior to downstream statistical analysis. A non-parametric Wilcoxon exact test was used to statistically compare the taxonomic composition in any two metagenomes.

Additionally, within MG-RAST, the functional annotations (hits to SEED Subsystems) were extracted (e-value less than 1 × 10^-5 ^and a sequence match length greater than 50 nucleotides) to compare functional attributes across these gut metagenomes. In order to identify statistically significant and biologically meaningful differences between the swine gut and other endiobiotic microbiomes, we employed the two-way Fisher's exact test with a Benjamin-Hochberg FDR multiple test correction within STAMP v1.0.2 [39].

### Diversity Indices

Observed richness, Chao1 estimator, abundance-based coverage estimator (ACE), jackknife estimator, and bootstrap estimator were used to evaluate community richness. Community diversity was described using Shannon, non-parametric Shannon, and Simpson indices within Mothur v 1.5.0 [40]. Sampling coverage was calculated using Good's coverage for the given operational taxonomic unit (OTU) definition, while the Boneh estimate was used to calculate the number of additional OTUs that would be observed for an additional 500 SSU reads. The aforementioned rRNA diversity indices and rarefaction curves were calculated using Mothur v 1.5.0 program with default parameters [40] and calculations for each index can found in the Mothur manual (http://www.mothur.org/wiki/Mothur_manual). Functional diversity was assessed using SEED Subsystems [41], COG, and Pfam abundances from all available gut metagenomes. Diversity estimators used included Shannon-Weiner, Simpson's lambda, and Pielou's evenness analyses for measuring species richness and evenness. Functional diversity estimates, K- dominance plots, Principal Components Analysis, and clustering were performed using the PRIMER-E ecological software package [42].

## Authors' contributions

RL carried out sample collection, sample processing, bioinformatic analyses, and manuscript preparation. JSD conceived of the study, and participated in its design and coordination and helped to draft the manuscript. SG participated in bioinformatic and statistical analyses. JM participated in bioinformatic analyses. DBO participated in design and coordination of the study and helped revise the manuscript. All authors read and approved the final manuscript.

## Supplementary Material

Additional file 1 **Figures S1-S13**. **Fig. S1**. Taxonomic distribution of viral sequences from swine feces. The percent of viral sequences retrieved from swine fecal GS20 (A) and FLX (B) metagenomes. Using the "Phylogenetic Analysis" tool within MG-RAST, the GS20 and FLX sequencing runs were searched against the SEED database using the BLASTx algorithm. The e-value cutoff for a hit to the database was 1×10^-5 ^with a minimum alignment length of 30 bp. **Fig. S2. **Taxonomic distribution of bacterial orders from swine and other currently available gut microbiomes within MG-RAST. The percent of sequences assigned to each bacterial order from swine and other gut metagenomes is shown. Using the "Phylogenetic Analysis" tool within MG-RAST, each gut metagenome was searched against the RDP and greengenes databases using the BLASTn algorithm. The percentage of each bacterial order from swine, human infant, and human adult metagenomes were each averaged since there was more than one metagenome for each of these hosts within the MG-RAST database. The e-value cutoff for 16S rRNA gene hits to the RDP and greengenes databases was 1×10^-5 ^with a minimum alignment length of 50 bp. **Fig. S3. **Taxonomic composition of bacterial genera using 16S rDNA sequences retrieved from swine fecal metagenomes. The percent of sequences assigned to each of the bacterial genera from the pig fecal GS20 (A) and FLX (B) metagenomes is shown. Using the "Phylogenetic Analysis" tool within MG-RAST, the GS20 and FLX pig fecal metagenomes were searched against the RDP and greengenes databases using the BLASTn algorithm. The e-value cutoff for 16S rRNA gene hits to the databases was 1×10^-5 ^with a minimum alignment length of 50 bp. **Fig. S4. **Dominance profiles of swine and other gut metagenomes available within MG-RAST. K-dominance plots were calculated based on the abundance of gut metagenomic sequences assigned at the RDP genus level taxonomy using the "Phylogenetic Analysis" tool within MG-RAST. The e-value cutoff for 16S rRNA gene hits to the RDP database was 1×10^-5 ^with a minimum alignment length of 50 bp. K-dominance for each of the individual gut metagenomes was calculated using PRIMER-E v6 software [42]. **Fig. S5. **Rarefaction curves for 16S rRNA gene sequences from swine and other gut metagenomes. Rarefaction curves were calculated based on the observed abundance of gut metagenomic sequences assigned at the RDP genus level taxonomy using MG-RAST's "Phylogenetic Analysis" tool. The e-value cutoff for 16S rRNA gene hits to the RDP database was 1×10^-5 ^with a minimum alignment length of 50 bp. Rarefaction curves for each gut metagenome were calculated within Mothur v 1.5.0 software using default parameters [40]. Rarefaction curves provide a way of comparing the richness observed in these different gut metagenomic samples. **Fig. S6. **Functional composition of the swine fecal microbiome. The percent of GS20 (A) and FLX (B) swine fecal metagenomic sequences assigned to general SEED Subsystems is shown. Using the "Metabolic Analysis" tool within MG-RAST, the GS20 and FLX pig fecal sequencing runs were searched against the SEED database using the BLASTx algorithm. The e-value cutoff for metagenomics sequence matches to the SEED Subsystem database was 1×10^-5 ^with a minimum alignment length of 30 bp. **Fig. S7. **Comparison of functional composition of swine and other currently available gut metagenomes within the MG-RAST pipeline. Percentage of gut metagenomic sequences assigned to general SEED Subsystems is shown. Using the "Metabolic Analysis" tool within MG-RAST, gut metagenomes were searched against the SEED database using the BLASTx algorithm. The percentage of each general SEED Subsystem from swine, human infant, and human adult metagenomes were each averaged since there was more than one metagenome for each of these hosts within the MG-RAST database. The e-value cutoff for metagenomic sequence matches to the SEED Subsystem database was 1×10^-5 ^with a minimum alignment length of 30 bp. **Fig. S8. **Percent distribution of prophage and DNA recombination genes from gut metagenomes available within the MG-RAST pipeline. Using the "Metabolic Analysis" tool within MG-RAST, the available gut metagenomes were searched against the SEED database using the BLASTx algorithm. Percentage contribution of each gut metagenome assigned to functional classes within "Prophage/DNA recombination" SEED Subsystem is shown. The e-value cutoff for metagenomic sequences matches to this SEED Subsystem was 1×10^-5 ^with a minimum alignment length of 30 bp. **Fig. S9. **Hierarchical clustering of gut metagenomes available within MG-RAST based on the relative abundance of cell wall and capsule genes. A matrix consisting of the number of reads assigned to genes within the "Cell wall and Capsule" SEED Subsystem from each gut metagenome was generated using the "Metabolic Analysis" tool within MG-RAST. The e-value cutoff for metagenomic sequences matches to this SEED Subsystem was 1×10^-5 ^with a minimum alignment length of 30 bp. Resemblance matrices were calculated using Bray- Curtis dissimilarities within PRIMER v6 software [41]. Clustering was performed using the complete linkage algorithm. Dotted branches denote that no statistical difference in similarity profiles could be identified for these respective nodes, using the SIMPROF test within PRMERv6 software. **Fig. S10. **Transposases derived from gut metagenomes available within JGI's IMG/M database. The percent of total annotated tranposase gene families from pig, mouse, human, and termite gut metagenomes is shown. The percentage of each transposase family from swine, human, and mouse gut metagenomes were each averaged since there was more than one metagenome for each of these hosts within the JGI's IMG/M database. Metagenomic sequences were assigned to transposase gene families using the IMG 2.8 pipeline. **Fig. S11. **Composition of resistance genes present with the swine fecal metagenome. The percent of swine fecal metagenomic sequences assigned to the "Resistance to Antibiotics and Toxic Compounds" SEED Subsystem is shown. The number of GS20 and FLX assigned to genes within this SEED Subsystem were combined. The e-value cutoff for metagenomic sequence matches to this SEED Subsystem database was 1×10^-5 ^with a minimum alignment length of 30 bp. **Fig. S12. **Differential functions within the swine fecal metagenome. A list of significantly different SEED Subsystems and their relative abundance are shown for pair-wise comparisons of the pig fecal metagenome versus other available gut metagenomes within the MG-RAST database. A matrix of the abundance of sequences assigned to each SEED Subsystem from each gut metagenome was generated using the "Metabolic Analysis" tool in MG-RAST. The number of reads from each individual pig, human infant, and human adult metagenomes were each combined since there was more than one metagenome for each of these hosts within the MG-RAST database. The e-value cutoff for metagenomic sequence matches to SEED Subsystems was 1×10^-5 ^with a minimum alignment length of 30 bp. Pair-wise comparisons of pig fecal metagenomes versus (A) Lean Mouse cecum (B) Cow rumen (C) Fish gut (D) Termite gut (E) Chicken cecum (F) Human adult (G) Human infant gut metagenomes are shown. Fisher exact tests were employed using the Benjamin-Hochberg FDR multiple test correction to generate a list of significantly different SEED Subsystems using STAMP v1.0.2 software [39]. Significantly different SEED Subsystems with a q-value less than 1×10^-5 ^are shown. Significantly different SEED Subsystems from the pig fecal metagenome are shown in blue and all other gut metagenomes are shown in orange. **Fig. S13. **Comparison of lipid biosynthesis genes from gut metagenomes available within the MG-RAST pipeline. Using the "Metabolic Analysis" tool within MG-RAST, the gut metagenomes were searched against the SEED database using the BLASTx algorithm. Percentage of gut metagenomic reads assigned to genes in the "Fatty Acid and Lipid Biosynthesis" SEED Subsystem is shown. The e-value cutoff for metagenomics sequence matches to this SEED Subsystem database was 1×10^-5 ^with a minimum alignment length of 30 bp. Click here for file

Additional file 2 **Tables S1-S6**. **Table S1**. The results of a Wilcoxon test to compare taxonomic distribution of bacterial orders from endobiotic microbiomes. **Table S2. **Binomial test for comparing abundance of bacteria phyla from distal gut metagenomes. **Table S3. **Binomial test for comparing abundance of bacteria genera from distal gut metagenomes. **Table S4. **Diversity analyses for endobiotic metagenomes using SEED Subsystem annotations. **Table S5. **Diversity analyses for endobiotic metagenomes using COG and Pfam annotations. **Table S6. **Pfams and COGs unique to swine fecal metagenomes. Click here for file
