# Supplementary material for: Comparative fecal metagenomics unveils unique functional capacity of the swine gut
Source: BMC Microbiol. 2011 May 15;11:103. doi: 10.1186/1471-2180-11-103 (PMC3123192; doi:10.1186/1471-2180-11-103)
Supplement: Additional file 1 — Figures S1-S13. Fig. S1. Taxonomic distribution of viral sequences from swine feces. The percent of viral sequences retrieved from swine fecal GS20 (A) and FLX (B) metagenomes. Using the "Phylogenetic Analysis" tool within MG-RAST, the GS20 and FLX sequencing runs were searched against the SEED database using the BLASTx algorithm. The e-value cutoff for a hit to the database was 1×10^-5^ with a minimum alignment length of 30 bp. Fig. S2. Taxonomic distribution of bacterial orders from swine and other currently available gut microbiomes within MG-RAST. The percent of sequences assigned to each bacterial order from swine and other gut metagenomes is shown. Using the "Phylogenetic Analysis" tool within MG-RAST, each gut metagenome was searched against the RDP and greengenes databases using the BLASTn algorithm. The percentage of each bacterial order from swine, human infant, and human adult metagenomes were each averaged since there was more than one metagenome for each of these hosts within the MG-RAST database. The e-value cutoff for 16S rRNA gene hits to the RDP and greengenes databases was 1×10^-5^ with a minimum alignment length of 50 bp. Fig. S3. Taxonomic composition of bacterial genera using 16S rDNA sequences retrieved from swine fecal metagenomes. The percent of sequences assigned to each of the bacterial genera from the pig fecal GS20 (A) and FLX (B) metagenomes is shown. Using the "Phylogenetic Analysis" tool within MG-RAST, the GS20 and FLX pig fecal metagenomes were searched against the RDP and greengenes databases using the BLASTn algorithm. The e-value cutoff for 16S rRNA gene hits to the databases was 1×10^-5^ with a minimum alignment length of 50 bp. Fig. S4. Dominance profiles of swine and other gut metagenomes available within MG-RAST. K-dominance plots were calculated based on the abundance of gut metagenomic sequences assigned at the RDP genus level taxonomy using the "Phylogenetic Analysis" tool within MG-RAST. The e-value cutoff for 16S rRNA gene hits [file 1471-2180-11-103-S1.DOC]

**Additional File 1, Supplemental Figures S1-S13**

A. B.

**
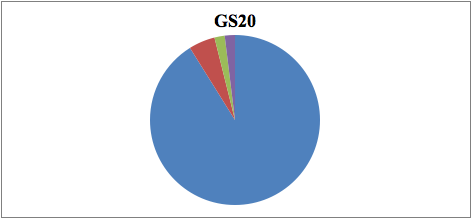

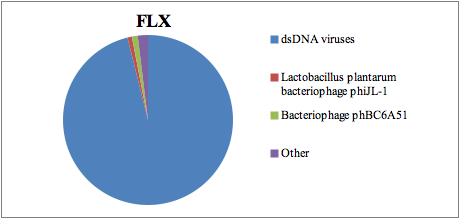
**

**Additional File 1, Fig. S1.**


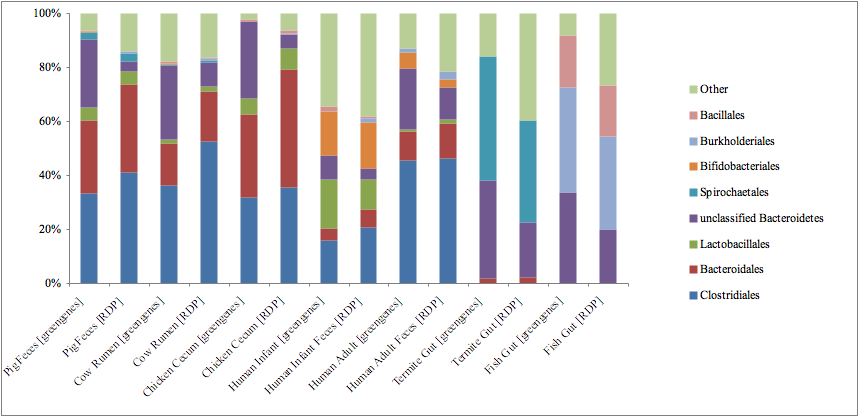


**Additional File 1, Fig. S2.**

A. B.


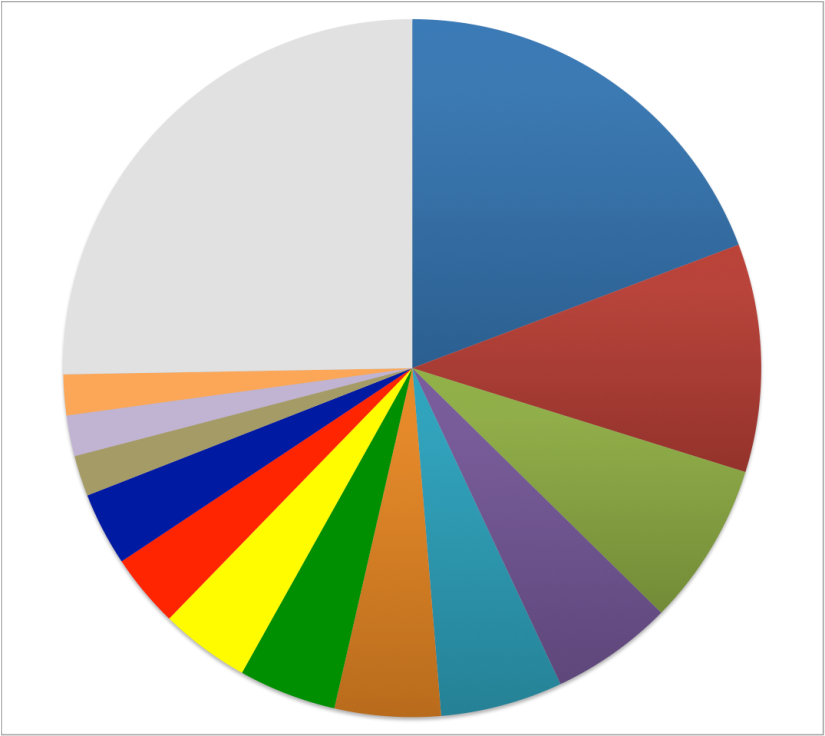

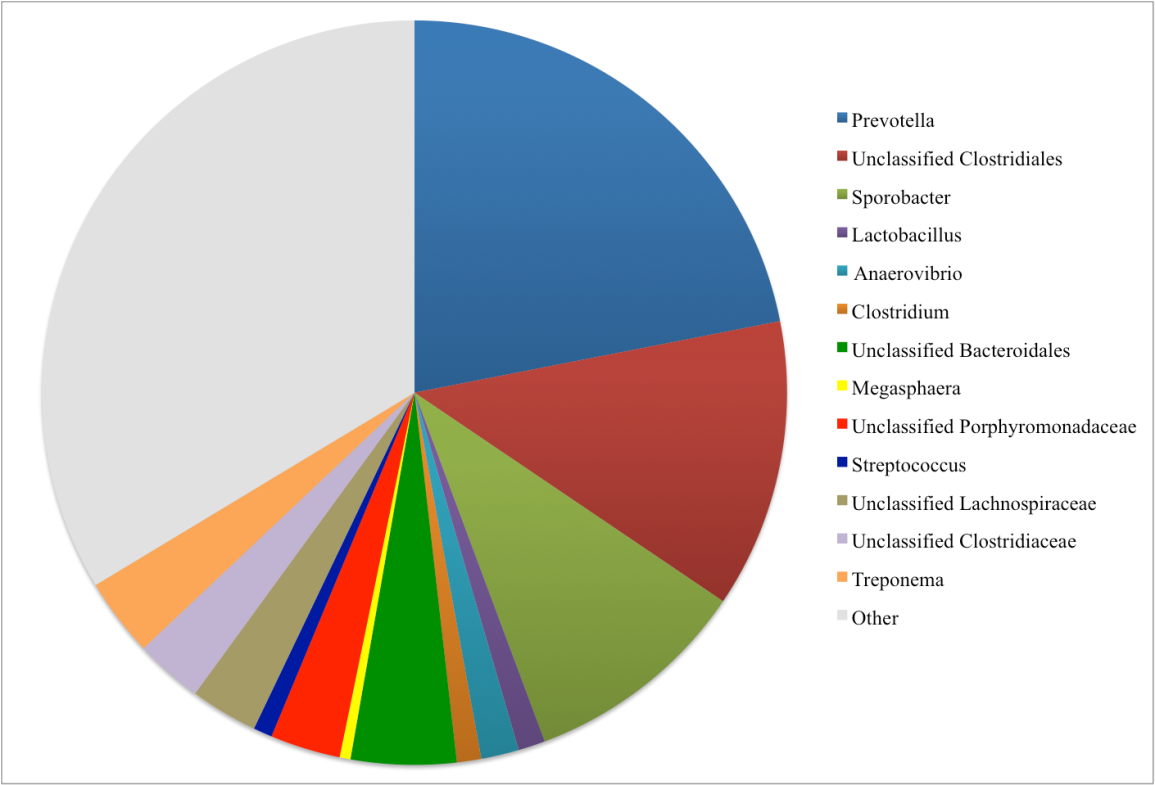


**Additional File 1, Fig. S3.**

**
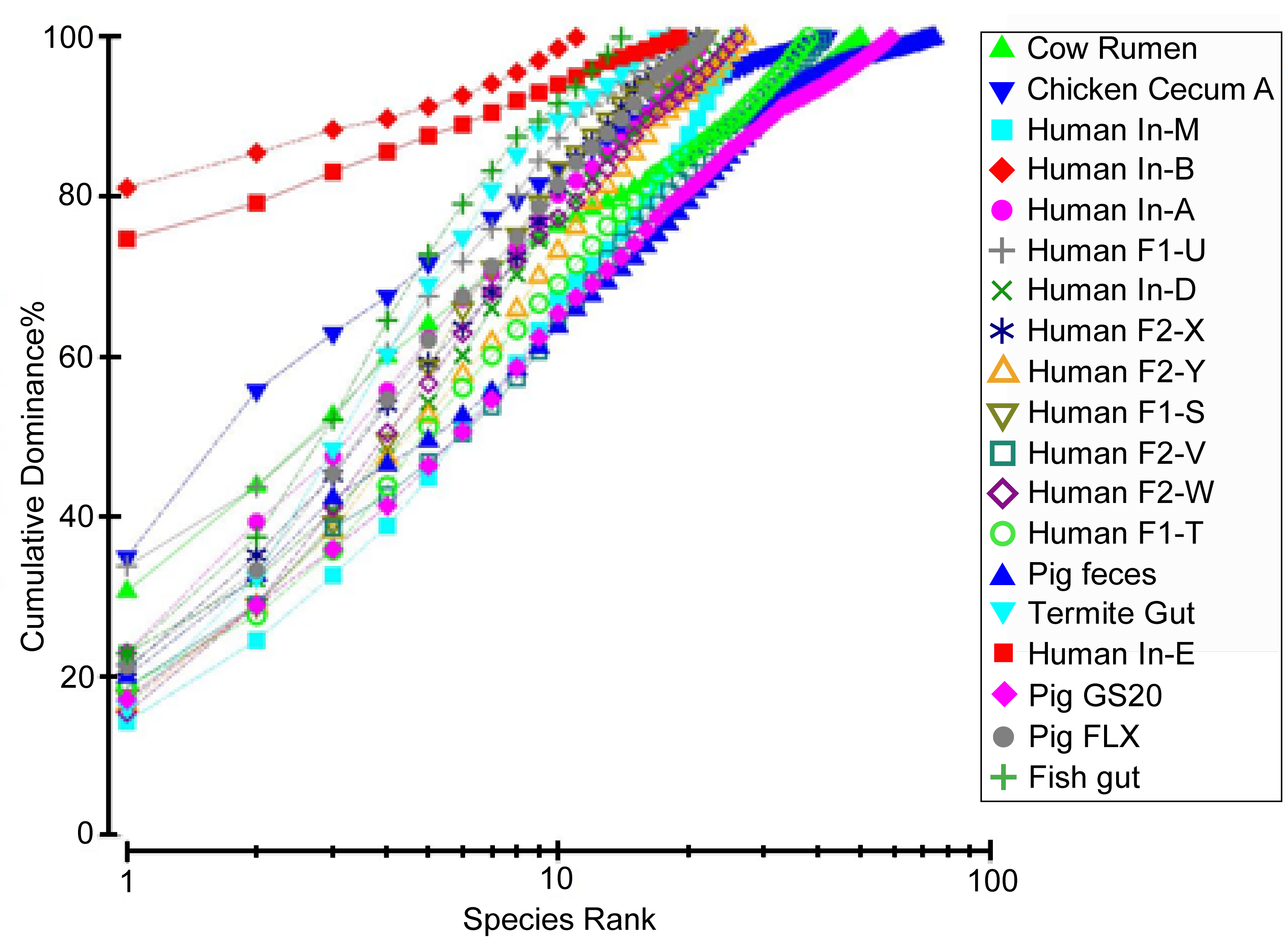
**

**Additional File 1, Fig. S4.**


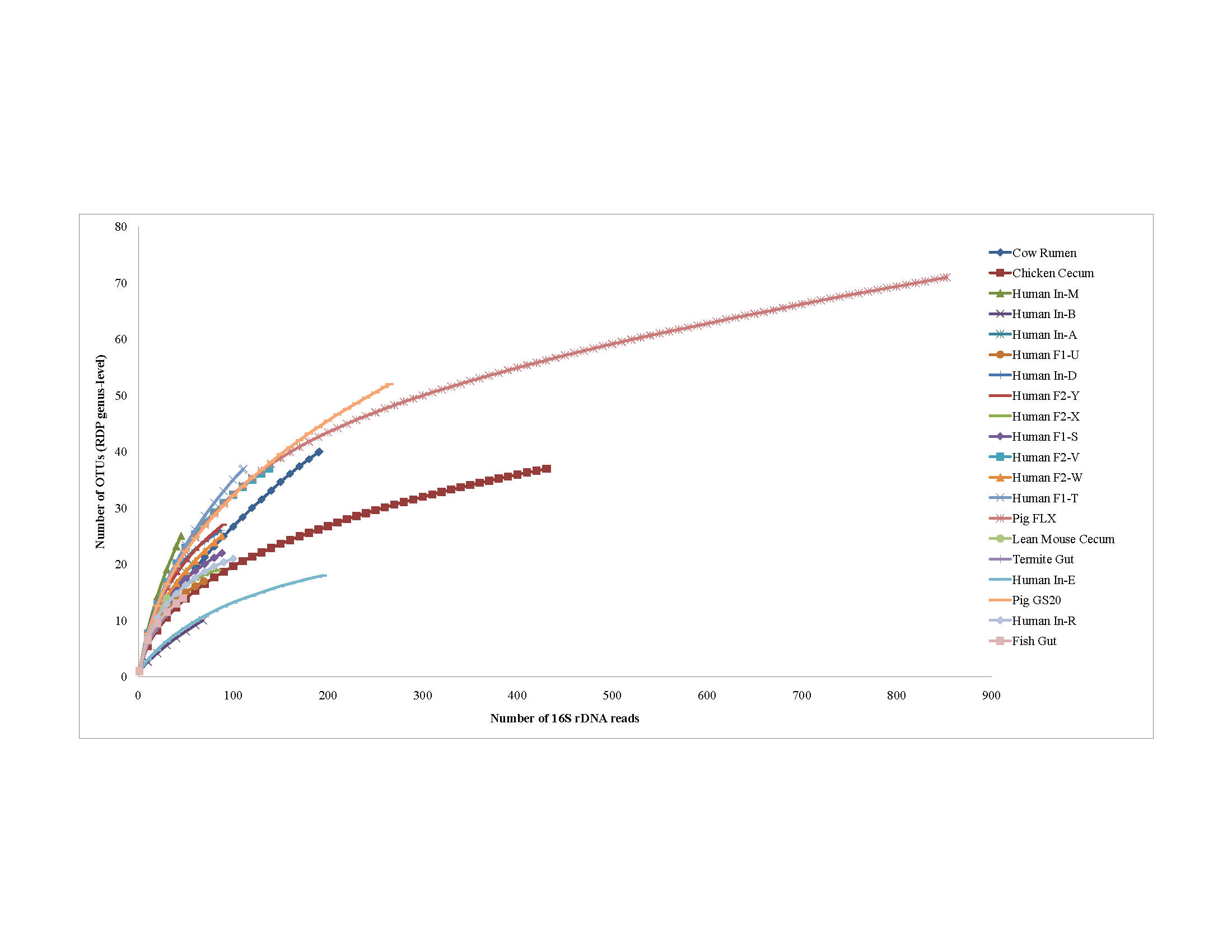


**Additional File 1, Fig. S5.**

A. B.

**
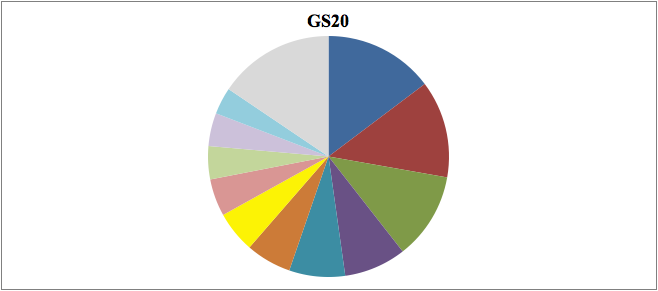

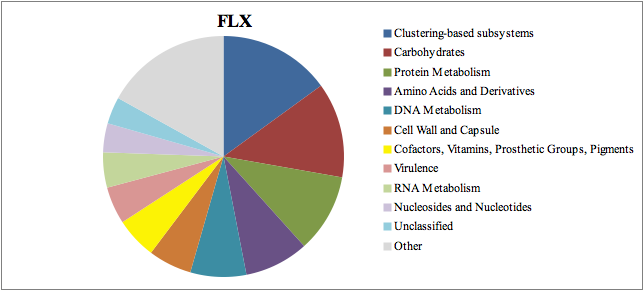
**

**Additional File 1, Fig. S6.**


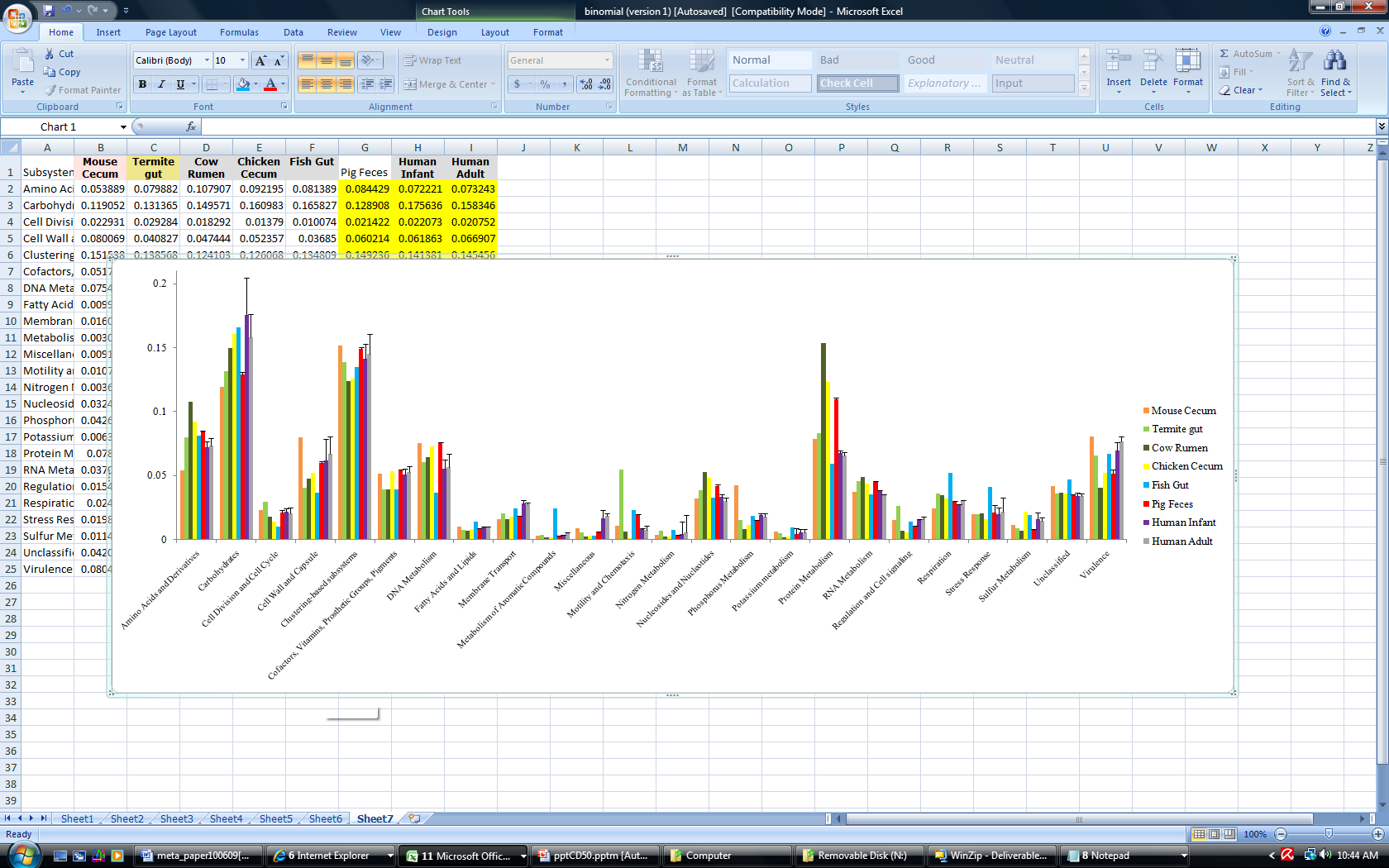


**Additional File 1, Fig. S7.**

B.


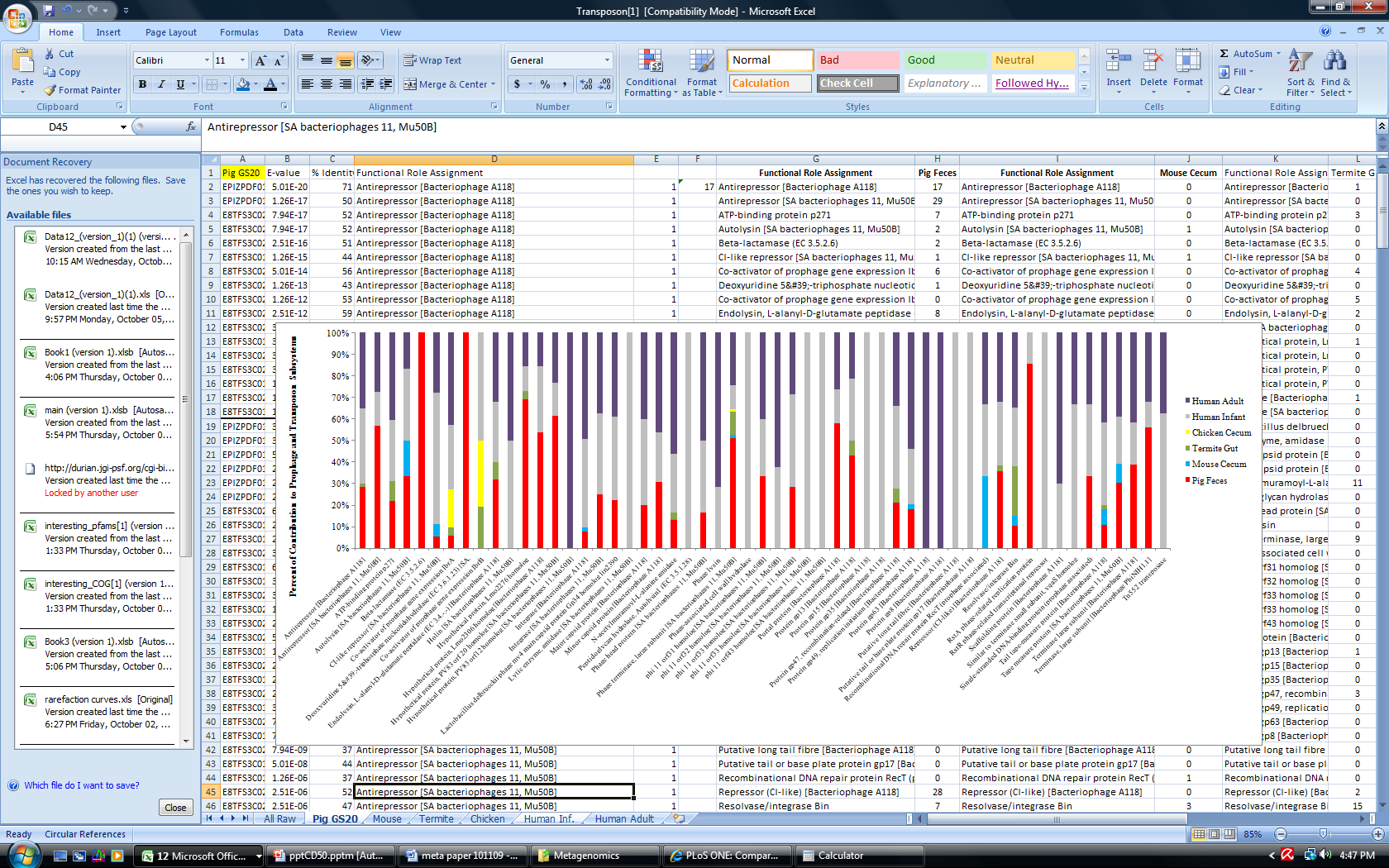


**Additional File 1, Fig. S8.**


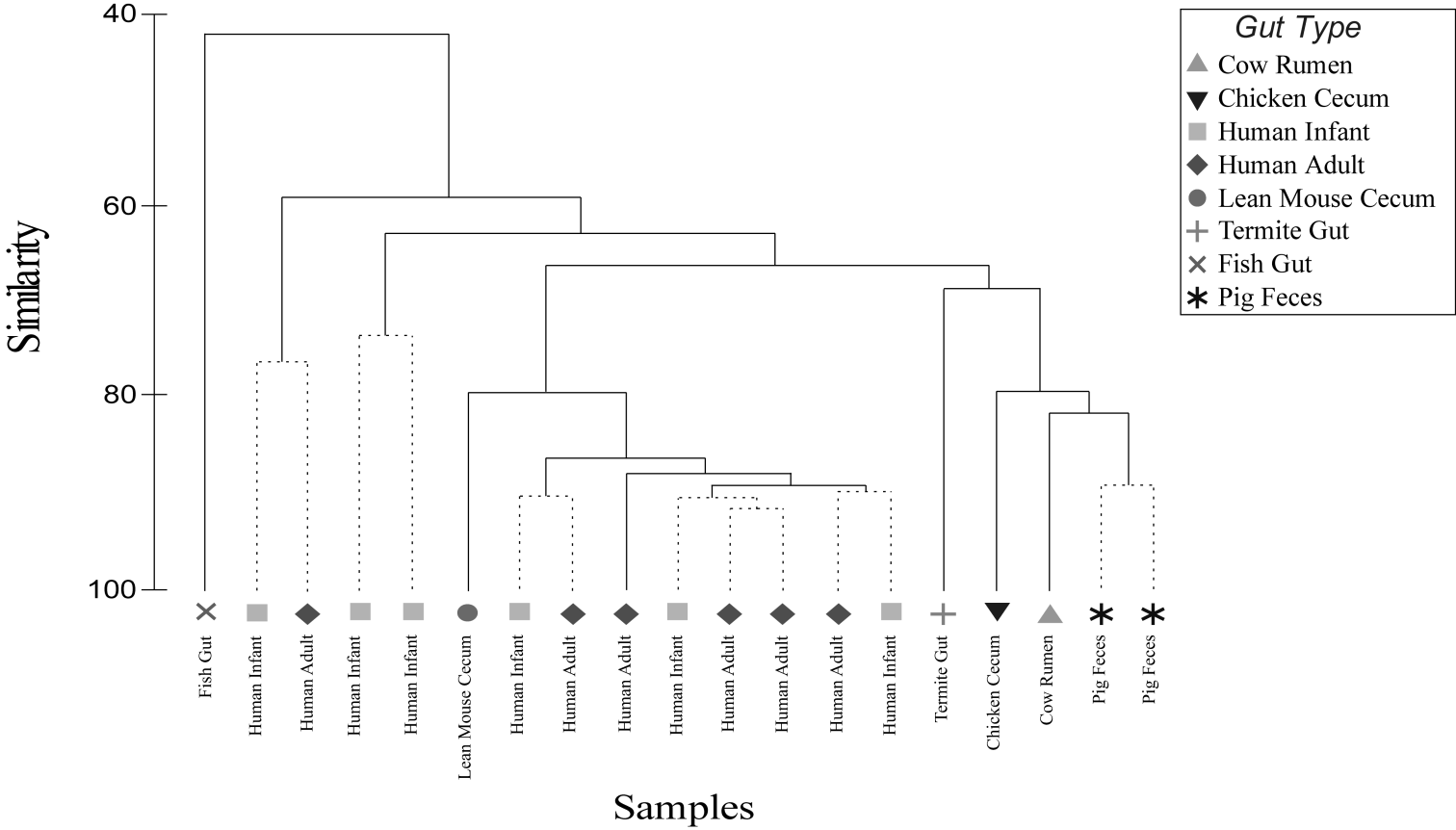


**Additional File 1, Fig. S9.**

**
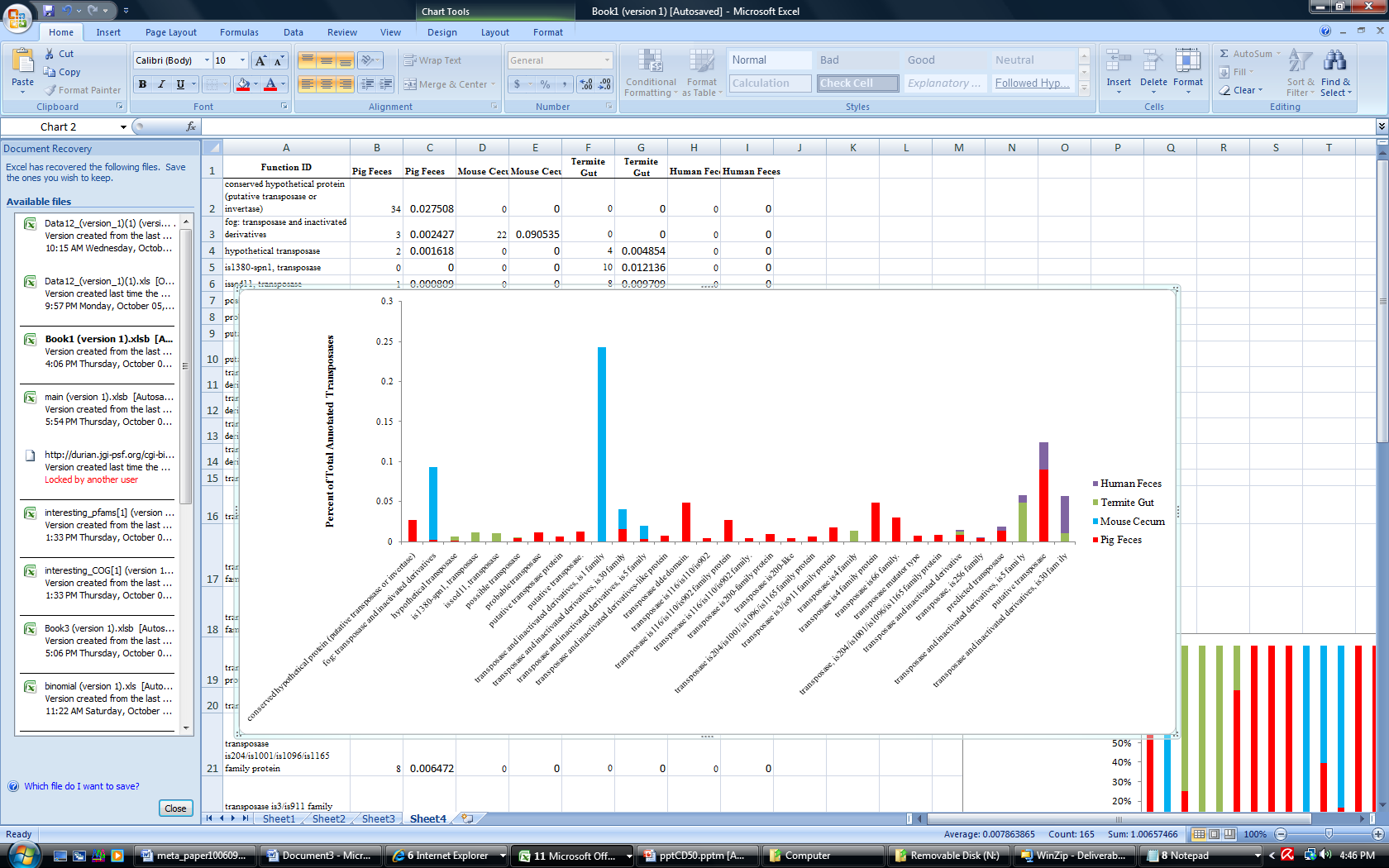
**

**Additional File 1, Fig. S10.**

**
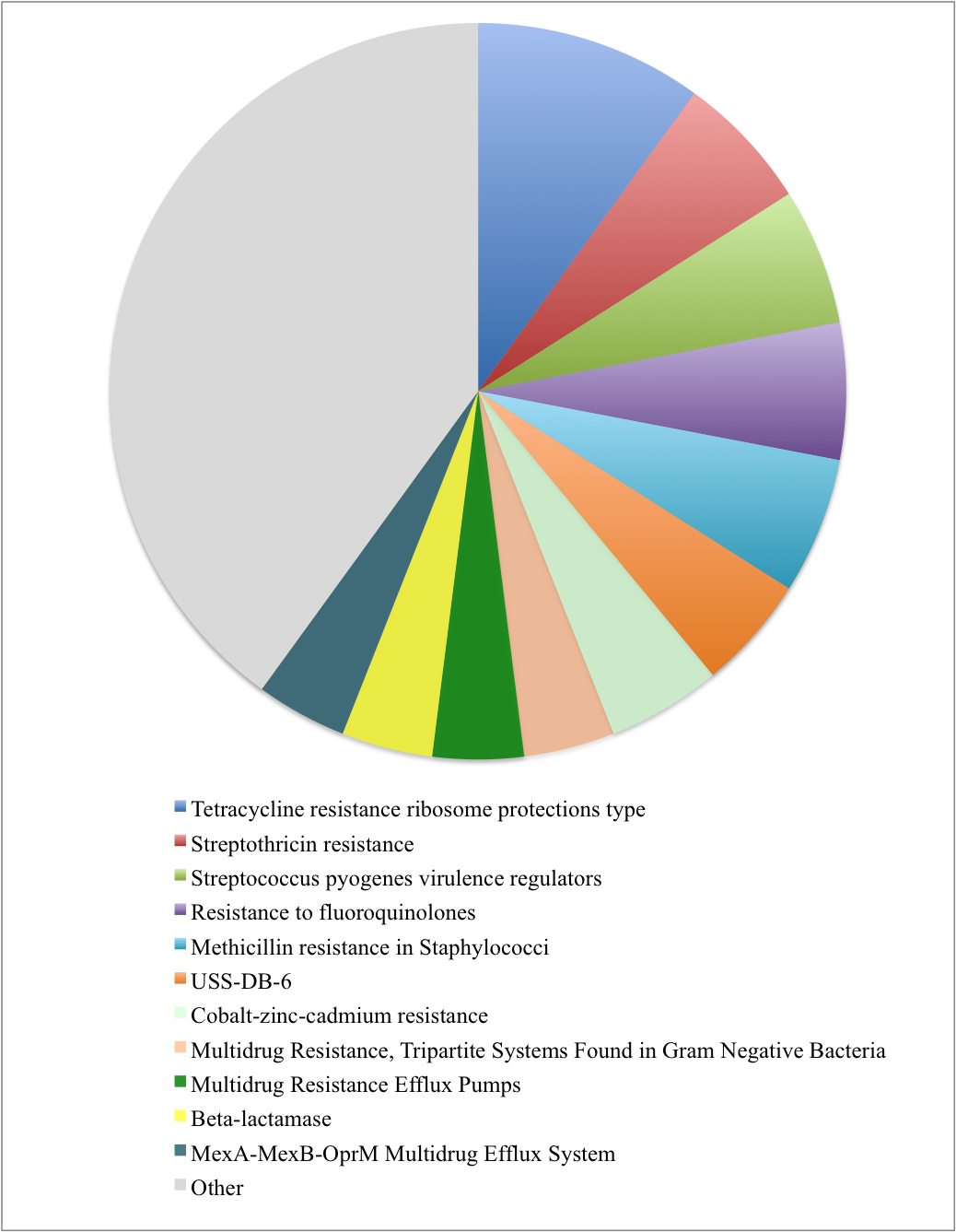
**

**Additional File 1, Fig. S11.**

A.


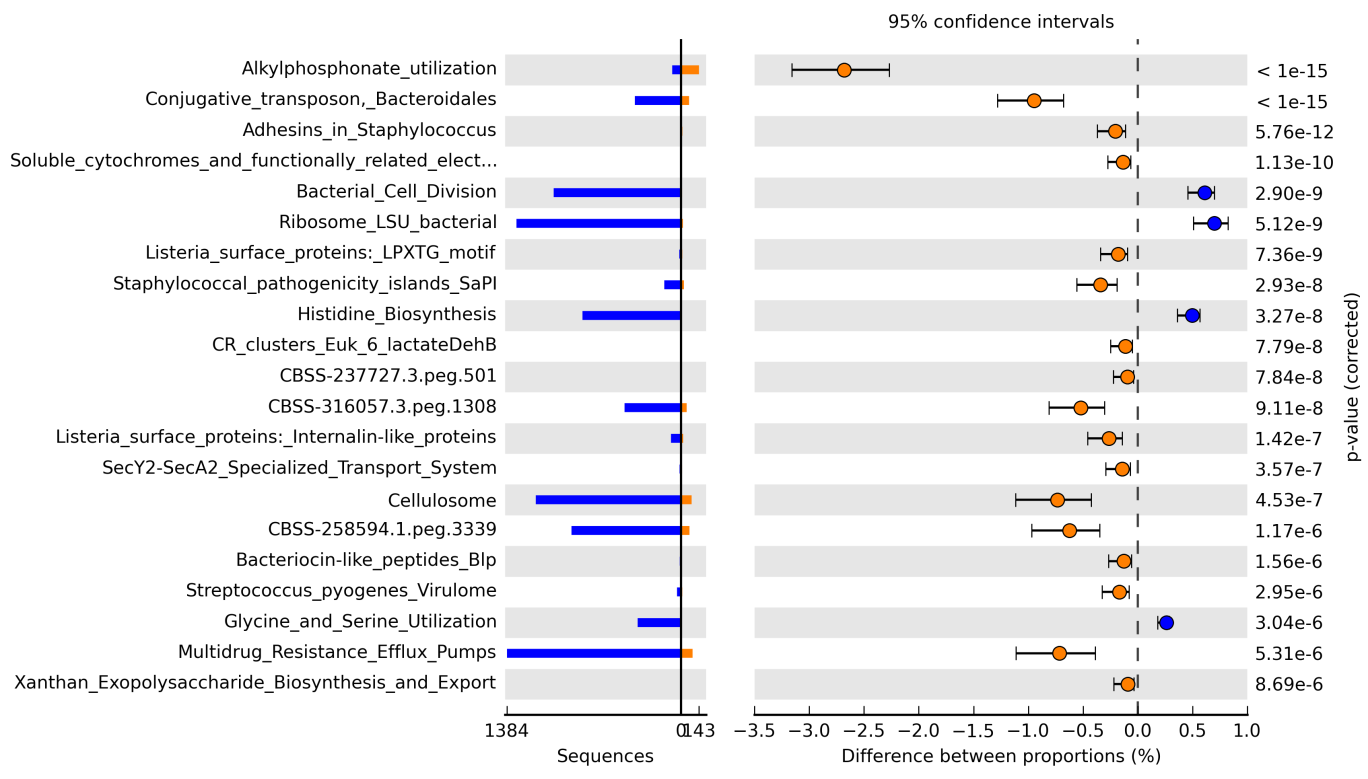


**Additional File 1, Fig. S12.**

B.

**
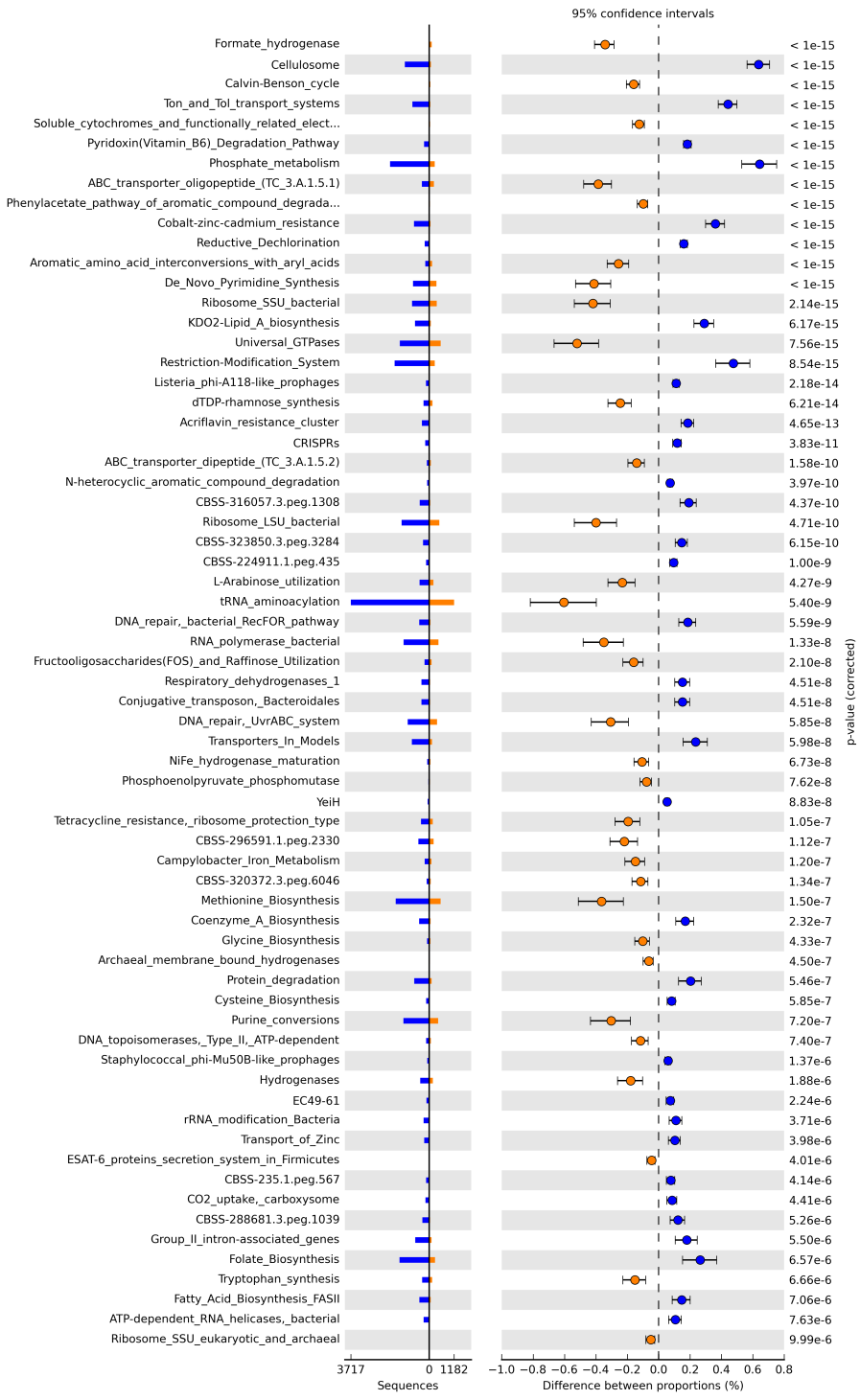
**

**Additional File 1, Fig. S12.**

C.


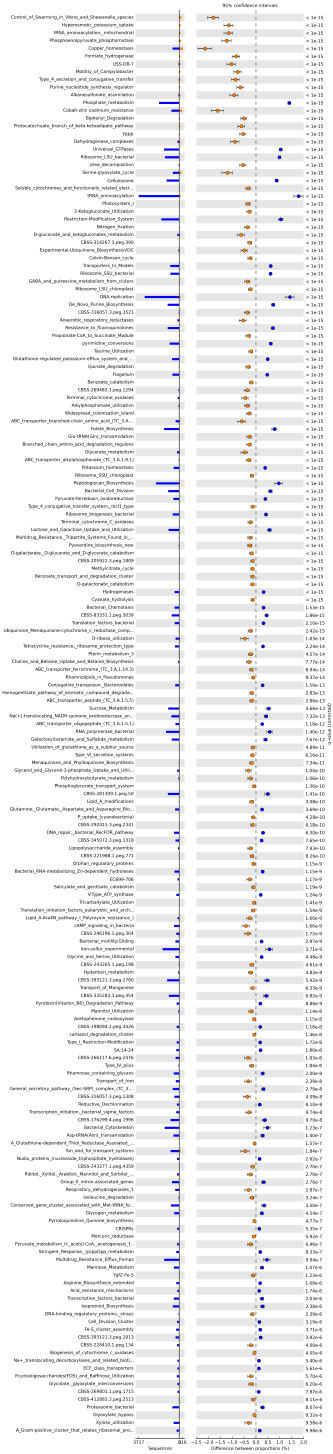

**Additional File 1, Fig. S12.**

D.


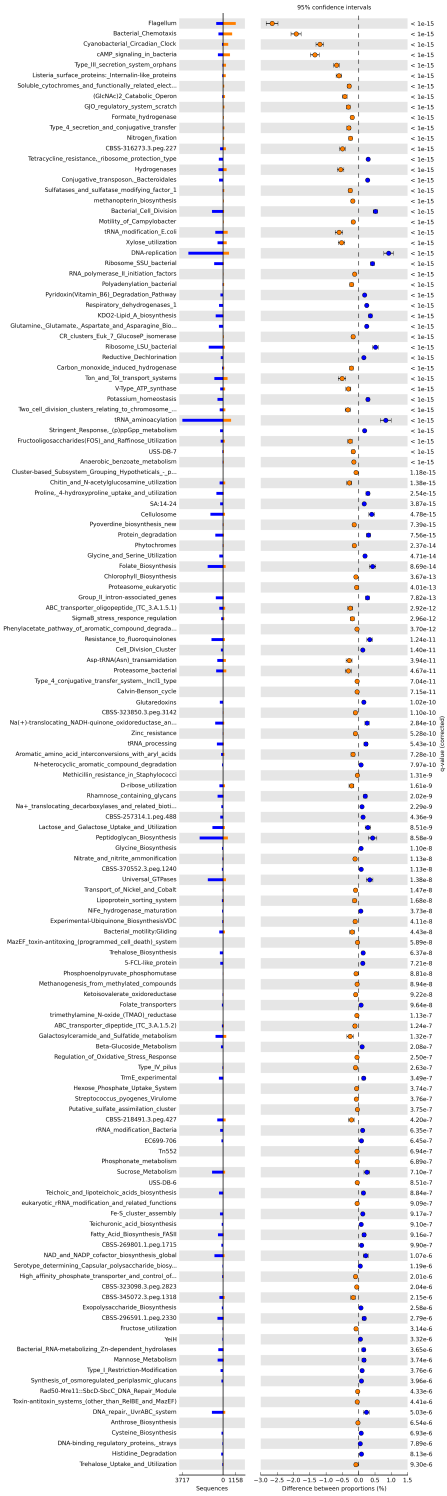


**Additional File 1, Fig. S12.**

E.


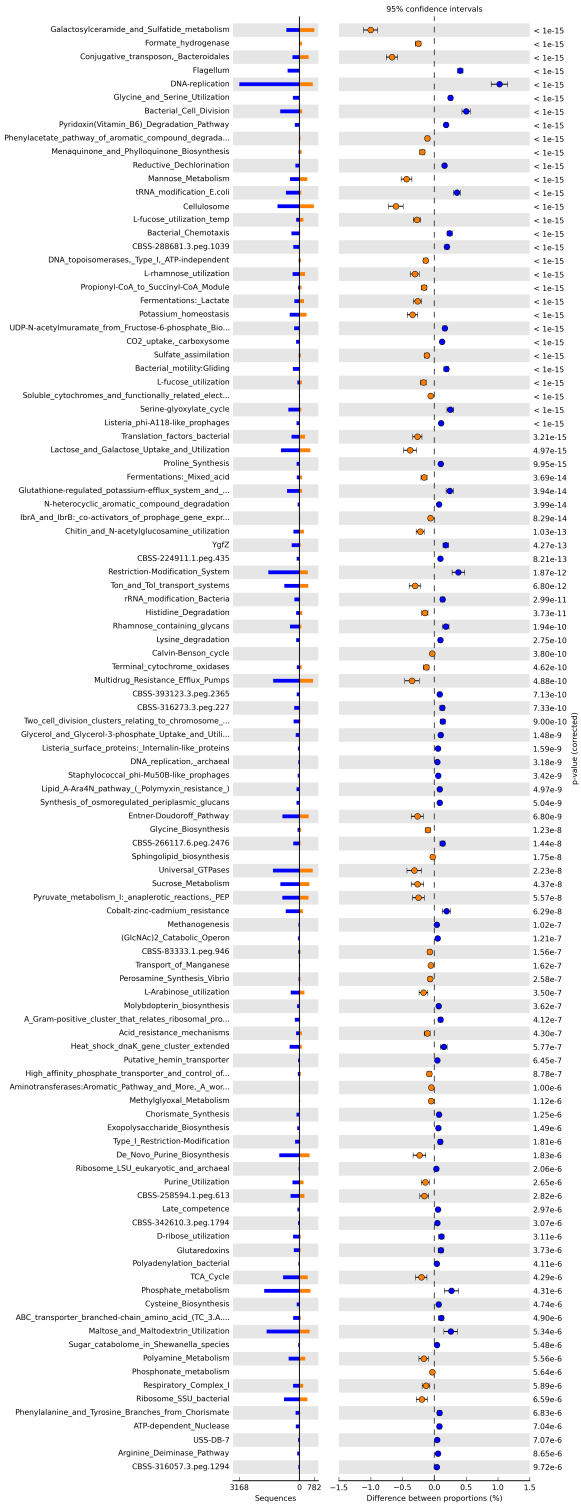


**Additional File 1, Fig. S12.**

F.


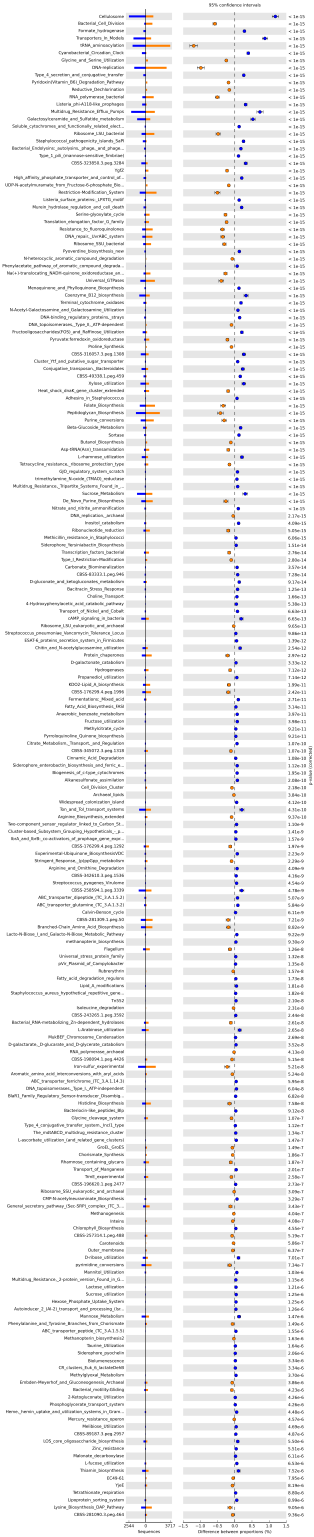


**Additional File 1, Fig. S12.**

G.


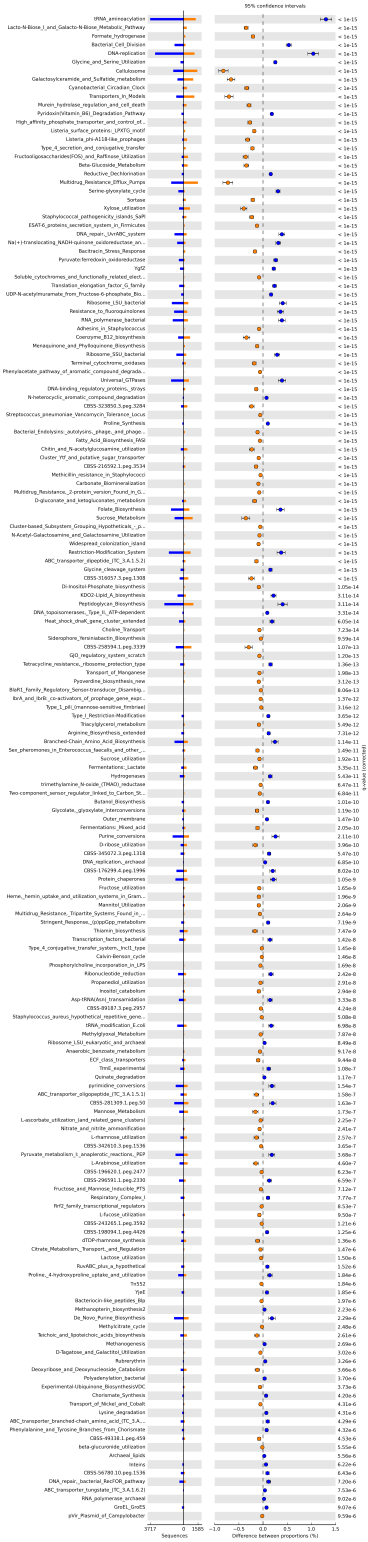


**Additional File 1, Fig. S12.**

**
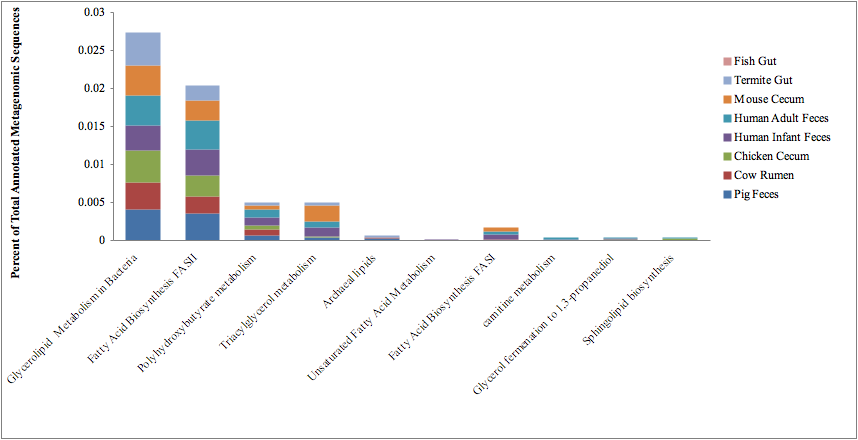
**

**Additional File 1, Fig. S13.**
