# Supplementary material for: Comparative fecal metagenomics unveils unique functional capacity of the swine gut
Source: BMC Microbiol. 2011 May 15;11:103. doi: 10.1186/1471-2180-11-103 (PMC3123192; doi:10.1186/1471-2180-11-103)
Supplement: Additional file 2 — Tables S1-S6. Table S1. The results of a Wilcoxon test to compare taxonomic distribution of bacterial orders from endobiotic microbiomes. Table S2. Binomial test for comparing abundance of bacteria phyla from distal gut metagenomes. Table S3. Binomial test for comparing abundance of bacteria genera from distal gut metagenomes. Table S4. Diversity analyses for endobiotic metagenomes using SEED Subsystem annotations. Table S5. Diversity analyses for endobiotic metagenomes using COG and Pfam annotations. Table S6. Pfams and COGs unique to swine fecal metagenomes. [file 1471-2180-11-103-S2.DOC]

**Additional File 2, Supplementary Tables 1-6**

**Additional File 2, Table S1.** The results of a Wilcoxon test to compare taxonomic distribution of bacterial orders from endobiotic microbiomes.

| **Metagenome** | **Z-score** | **One-sided p value** | **Two-sided p value** |
| --- | --- | --- | --- |
| **Cow Rumen** | 1.8544 | 0.0318 | 0.0637 |
| **Chicken Cecum** | 2.6024 | 0.0046 | 0.0093 |
| **Human In-A** | 2.5245 | 0.0058 | 0.0116 |
| **Human In-B** | 3.7919 | 0.0001 | 0.0002 |
| **Human In-D** | 2.1297 | 0.0166 | 0.0332 |
| **Human In-E** | 2.8309 | 0.0023 | 0.0046 |
| **Human In-M** | 2.76 | 0.0029 | 0.0058 |
| **Human In-R** | 2.35 | 0.0094 | 0.0188 |
| **Human F1-S** | 3.2413 | 0.0059 | 0.0012 |
| **Human F1-T** | 1.6986 | 0.0447 | 0.0894 |
| **Human F1-U** | 3.1582 | 0.0008 | 0.0016 |
| **Human F2-V** | 2.0933 | 0.0182 | 0.0363 |
| **Human F2-W** | 2.4777 | 0.0066 | 0.0132 |
| **Human F2-X** | 2.8621 | 0.0021 | 0.0042 |
| **Human F2-Y** | 2.54 | 0.0055 | 0.1102 |
| **Mouse Cecum** | 3.4127 | 0.0003 | 0.0006 |
| **Termite Gut** | 3.4543 | 0.0003 | 0.0006 |
| **Fish gut** | 3.7503 | 0.0001 | 0.0002 |

**Additional File 2, Table S2.** Binomial test for comparing abundance of bacteria phyla from distal gut metagenomes.

|  | **p-value** | **Statistical outcome** |
| --- | --- | --- |
| **Cow Rumen vs Pig Feces** | | |
| *Firmicutes* | 3.17E-05 | Less abundant in Pig Feces |
| *Bacteroidetes* | 1.39E-03 | More abundant in Pig Feces |
| *Actinobacteria* | 2.56E-03 | Less abundant in Pig Feces |
| *Proteobacteria* | 3.15E-02 | More abundant in Pig Feces |
| *Spirochaetes* | 3.68E-02 | More abundant in Pig Feces |
| **Chicken Cecum vs Pig Feces** | | |
| *Spirochaetes* | 2.78E-08 | More abundant in Pig Feces |
| *Bacteroidetes* | 1.83E-04 | Less abundant in Pig Feces |
| *Proteobacteria* | 4.98E-04 | More abundant in Pig Feces |
| *Fibrobacteres* | 1.30E-02 | More abundant in Pig Feces |
| **Human Infant vs Pig Feces** | | |
| *Actinobacteria* | 1.69E-57 | Less abundant in Pig Feces |
| *Bacteroidetes* | 5.32E-10 | More abundant in Pig Feces |
| *Spirochaetes* | 2.78E-02 | More abundant in Pig Feces |
| **Human Adult vs Pig Feces** | | |
| *Actinobacteria* | 7.37E-09 | Less abundant in Pig Feces |
| *Bacteroidetes* | 1.25E-03 | More abundant in Pig Feces |
| *Proteobacteria* | 3.72E-03 | Less abundant in Pig Feces |
| *Spirochaetes* | 1.44E-02 | More abundant in Pig Feces |
| **Fish Gut vs Pig Feces** | | |
| *Proteobacteria* | 8.95E-24 | Less abundant in Pig Feces |
| *Bacteroidetes* | 1.02E-10 | More abundant in Pig Feces |
| **Termite Gut vs Pig Feces** | | |
| *Spirochaetes* | 5.89E-25 | Less abundant in Pig Feces |
| *Firmicutes* | 1.12E-09 | More abundant in Pig Feces |
| *Bacteroidetes* | 1.10E-08 | More abundant in Pig Feces |
| *Actinobacteria* | 1.57E-05 | Less abundant in Pig Feces |
| *Fibrobacteres* | 1.15E-02 | Less abundant in Pig Feces |

**Additional File 2, Table S3.** Binomial test for comparing abundance of bacteria genera from distal gut metagenomes.

|  | **p-value** | **Statistical Outcome** |
| --- | --- | --- |
| **Cow rumen vs Pig Feces** | | |
| *Butyrivibrio* | 2.01E-03 | Less abundant in Pig Feces |
| *Anaerovibrio* | 2.89E-02 | More abundant in Pig Feces |
| *Treponema* | 1.64E-02 | More abundant in Pig Feces |
| **Chicken Cecum vs Pig Feces** | | |
| *Prevotella* | 4.69E-48 | More abundant in Pig Feces |
| *Bacteroides* | 5.89E-24 | Less abundant in Pig Feces |
| *Treponema* | 6.26E-03 | More abundant in Pig Feces |
| *Anaerovibrio* | 1.33E-02 | More abundant in Pig Feces |
| *Lactobacillus* | 1.33E-02 | Less abundant in Pig Feces |
| *Sporobacter* | 2.06E-02 | More abundant in Pig Feces |
| **Human Infant vs Pig Feces** | | |
| *Bifidobacterium* | 1.18E-28 | Less abundant in Pig Feces |
| *Prevotella* | 6.12E-05 | More abundant in Pig Feces |
| *Treponema* | 4.32E-02 | More abundant in Pig Feces |
| *Bacteroides* | 6.53E-04 | Less abundant in Pig Feces |
| **Human Adult vs Pig Feces** | | |
| *Prevotella* | 6.13E-03 | More abundant in Pig Feces |
| *Bifidobacterium* | 6.13E-03 | Less abundant in Pig Feces |
| *Treponema* | 1.63E-02 | More abundant in Pig Feces |
| *Bacteroides* | 1.57E-04 | Less abundant in Pig Feces |
| **Termite Gut vs Pig Feces** | | |
| *Treponema* | 1.69E-11 | More abundant in Pig Feces |
| *Spirochaeta* | 5.87E-10 | Less abundant in Pig Feces |
| *Prevotella* | 7.80E-03 | More abundant in Pig Feces |
| **Fish Gut vs Pig Feces** | | |
| *Paenibacillus* | 1.28E-09 | Less abundant in Pig Feces |
| *Herbaspirillum* | 1.87E-06 | Less abundant in Pig Feces |
| *Ralstonia* | 3.04E-03 | Less abundant in Pig Feces |
| *Prevotella* | 6.05E-03 | More abundant in Pig Feces |
| *Treponema* | 3.60E-02 | More abundant in Pig Feces |

**Additional File 2, Table S4.** **Diversity analyses for endobiotic metagenomes using SEED Subsystem annotations.**

|  | **S** | **N** | **d** | **J'** | **Brillouin** | **Fisher** | **ES(5000)** |
| --- | --- | --- | --- | --- | --- | --- | --- |
| **Pig Feces GS20** | 574 | 16093 | 59.16 | 0.871 | 5.458 | 116.3 | 472 |
| **Pig Feces FLX** | 714 | 117061 | 61.09 | 0.851 | 5.575 | 101.2 | 488.3 |
| **Human In-A** | 570 | 11722 | 60.73 | 0.8865 | 5.527 | 125.3 | 505.4 |
| **Human In-B** | 461 | 5210 | 53.75 | 0.8813 | 5.25 | 122.1 | 456.8 |
| **Human In-D** | 623 | 21569 | 62.33 | 0.876 | 5.573 | 119.8 | 505.2 |
| **Human In-E** | 555 | 12022 | 58.97 | 0.8686 | 5.397 | 120.3 | 474.3 |
| **Human In-M** | 612 | 9802 | 66.48 | 0.8921 | 5.603 | 144.7 | 553.1 |
| **Human In-R** | 626 | 20477 | 62.96 | 0.8787 | 5.591 | 122.1 | 512 |
| **Human F1-S** | 609 | 17794 | 62.13 | 0.8797 | 5.567 | 122.1 | 507.2 |
| **Human F1-U** | 604 | 12009 | 64.19 | 0.8975 | 5.644 | 134 | 532.7 |
| **Human F2-V** | 659 | 24015 | 65.24 | 0.8757 | 5.623 | 125.3 | 522.8 |
| **Human F2-W** | 620 | 19007 | 62.83 | 0.8807 | 5.592 | 122.8 | 514.2 |
| **Human F2-X** | 629 | 18778 | 63.82 | 0.8805 | 5.602 | 125.4 | 519.5 |
| **Human F2-Y** | 617 | 21710 | 61.69 | 0.8718 | 5.538 | 118.2 | 505.7 |
| **Lean Mouse Cecum** | 496 | 5146 | 57.92 | 0.8906 | 5.359 | 135.4 | 493 |
| **Termite Gut** | 591 | 33635 | 56.6 | 0.8587 | 5.439 | 101.8 | 453.7 |

**Additional File 2, Table S5.** **Diversity analyses for endobiotic metagenomes using COG and Pfam annotations.**

| **Sample** | **S** | **N** | **D** | **J'** | **Brillouin** | **Fisher** | **ES(5000)** |
| --- | --- | --- | --- | --- | --- | --- | --- |
| **Yorkshire Pig Fecal Metagenome GS20 (COG)** | 2821 | 34010 | 270.3 | 0.9199 | 7.157 | 730.5 | 1672 |
| **Yorkshire Pig Fecal Metagenome GS20 (Pfam)** | 3043 | 50716 | 280.8 | 0.8785 | 6.936 | 710.7 | 1500 |
| **Yorkshire Pig Fecal Metagenome FLX (COG)** | 3717 | 97095 | 323.6 | 0.9081 | 7.385 | 766.5 | 1806 |
| **Yorkshire Pig Fecal Metagenome FLX (Pfam)** | 4314 | 141670 | 363.6 | 0.8574 | 7.115 | 840.4 | 1610 |
| **Human Gut Community Subject 7 (COG)** | 2256 | 12888 | 238.3 | 0.9332 | 6.932 | 791.7 | 1650 |
| **Human Gut Community Subject 7 (Pfam)** | 2065 | 13849 | 216.4 | 0.9078 | 6.692 | 671.9 | 1491 |
| **Human Gut Community Subject 8 (COG)** | 2295 | 15978 | 237 | 0.9243 | 6.919 | 734.4 | 1598 |
| **Human Gut Community Subject 8 (Pfam)** | 2116 | 19015 | 214.7 | 0.8962 | 6.675 | 609.5 | 1424 |
| **Termite Gut (COG)** | 2261 | 44458 | 211.2 | 0.9117 | 6.939 | 503.3 | 1410 |
| **Termite Gut (Pfam)** | 2079 | 49791 | 192.1 | 0.8809 | 6.645 | 438.5 | 1253 |
| **Mouse Gut Community lean1 (COG)** | 816 | 1562 | 110.8 | 0.9421 | 5.699 | 689.6 | 816 |
| **Mouse Gut Community lean1 (Pfam)** | 777 | 1637 | 104.9 | 0.9281 | 5.609 | 578.8 | 777 |
| **Mouse Gut Community lean2 (COG)** | 782 | 1424 | 107.6 | 0.9691 | 5.806 | 711.5 | 782 |
| **Mouse Gut Community lean 2 (Pfam)** | 637 | 1317 | 88.54 | 0.9134 | 5.333 | 485.7 | 637 |
| **Mouse Gut Community lean3 (COG)** | 787 | 1508 | 107.4 | 0.9144 | 5.493 | 664.2 | 787 |
| **Mouse Gut Community lean3 (Pfam)** | 749 | 1578 | 101.6 | 0.8989 | 5.394 | 558 | 749 |

**Additional File 2, Table S6. Pfams and COGs unique to swine fecal metagenomes.**

| **Contig Name** | **Contig Length** | **Number of Reads** | **Predicted Protein** | **Organism** | **Accession Number** | **E-value** | **Percent Identity** |
| --- | --- | --- | --- | --- | --- | --- | --- |
| **contig09884** | 1444 | 159 | hypothetical protein | *Bacteroides* *fragilis* | BAA95637 | 0 | 99% |
| **contig00095** | 646 | 22 | tetracycline resistant protein TetQ | *Bacteroides* sp. D1 | ZP 04543830 | 2.00E-111 | 99% |
| **contig01271** | 812 | 22 | tetracycline resistance protein | *Prevotella intermedia* | AAB51122 | 3.00E-102 | 98% |
| **contig01956** | 731 | 17 | macrolide-efflux protein | *Faecalibacterium prausnitzii* A2-165 | ZP 05613628 | 3.00E-85 | 99% |
| **contig01189** | 549 | 14 | macrolide-efflux protein | *Bacteroides* finegoldii DSM 17565 | ZP 05859238 | 8.00E-83 | 98% |
| **contig00070** | 603 | 11 | rRNA (guanine-N1-)-methyltransferase | *Faecalibacterium prausnitzii* A2-165 | ZP 05614052 | 2.00E-81 | 100% |
| **contig07794** | 846 | 27 | putative transposase | *Bacteroides* *fragilis* | AAA22911 | 4.00E-81 | 98% |
| **contig03360** | 671 | 10 | ABC transporter, ATP-binding protein | *Bacillus thuringiensis* serovar pondicheriensis BGSC 4BA1 | ZP 04090641 | 8.00E-77 | 77% |
| **contig09748** | 650 | 13 | hypothetical protein PRABACTJOHN 03572 | *Parabacteroides johnsonii* DSM 18315 | ZP 03477882 | 9.00E-71 | 77% |
| **contig00180** | 846 | 26 | macrolide-efflux protein | *Faecalibacterium prausnitzii* A2-165 | ZP 05613628 | 6.00E-67 | 90% |
| **contig00608** | 527 | 7 | ISPg3, transposase | *Prevotella* tannerae ATCC 51259 | ZP 05734821 | 1.00E-59 | 67% |
| **contig04843** | 578 | 7 | hypothetical protein COPEUT 02459 | *Coprococcus eutactus* ATCC 27759 | ZP 02207638 | 2.00E-57 | 88% |
| **contig00340** | 847 | 24 | conserved hypothetical protein | *Bacteroides* sp. 4 3 47FAA | ZP 05257903 | 6.00E-56 | 72% |
| **contig02245** | 616 | 7 | putative transposase | *Bacteroides* thetaiotaomicron VPI-5482 | NP 809147 | 3.00E-52 | 62% |
| **contig09776** | 531 | 9 | resolvase, N domain protein | *Faecalibacterium prausnitzii* A2-165 | ZP 05613620 | 5.00E-41 | 100% |
| **contig02310** | 557 | 11 | replication initiator protein A | *Faecalibacterium prausnitzii* A2-165 | ZP 05613624 | 1.00E-38 | 100% |
| **contig02075** | 524 | 9 | Transposase | *Bacteroides* *fragilis* 3 1 12 | ZP 05284372 | 7.00E-38 | 92% |
| **contig02837** | 529 | 7 | hypothetical protein CLOSS21 01510 | *Clostridium* sp. SS2/1 | ZP 02439046 | 6.00E-37 | 67% |
| **contig09732** | 632 | 11 | hypothetical protein BACCOP 00975 | *Bacteroides* coprocola DSM 17136 | ZP 03009123 | 1.00E-35 | 62% |
| **contig09862** | 574 | 16 | conserved hypothetical protein | *Oxalobacter formigenes* HOxBLS | ZP 04576182 | 1.00E-34 | 100% |
| **contig00069** | 897 | 21 | regulatory protein | *Sphingobacterium spiritivorum* ATCC 33300 | ZP 03965851 | 4.00E-29 | 43% |
| **contig00129** | 529 | 9 | transposase, putative | *Bacteroides* sp. 2 1 7 | ZP 05288481 | 8.00E-26 | 75% |
| **contig00130** | 674 | 11 | hypothetical protein BACCOP 00975 | *Bacteroides* coprocola DSM 17136 | ZP 03009123 | 6.00E-24 | 43% |
| **contig09924** | 1355 | 55 | conserved hypothetical protein | *Magnetospirillum gryphiswaldense* MSR-1 | CAJ30045 | 2.00E-23 | 45% |
| **contig00140** | 552 | 13 | ISPg7, transposase | *Cyanothece* sp. PCC 8802 | YP 003135760 | 5.00E-23 | 44% |
| **contig00572** | 675 | 16 | transposase, putative | *Bacteroides* sp. 2 1 7 | ZP 05288481 | 2.00E-21 | 57% |
| **contig09792** | 556 | 9 | hypothetical protein ALIPUT 01364 | *Alistipes putredinis* DSM 17216 | ZP 02425220 | 2.00E-16 | 67% |
| **contig09902** | 528 | 14 | putative transposase | *Lentisphaera araneosa* HTCC2155 | ZP 01873850 | 2.00E-12 | 63% |
| **contig09796** | 867 | 17 | hypothetical protein CLONEX 03424 | *Clostridium* nexile DSM 1787 | ZP 03291203 | 3.00E-07 | 35% |
| **contig01049** | 548 | 5 | No significant similarity found | - | - | - | - |
| **contig04775** | 565 | 4 | No significant similarity found | - | - | - | - |
| **contig09740** | 531 | 7 | No significant similarity found | - | - | - | - |
| **contig09927** | 656 | 29 | No significant similarity found | - | - | - | - |
